# Human-associated bacteria adopt an unusual route for synthesizing 3-acetylated tetramates for environmental adaptation

**DOI:** 10.1186/s40168-023-01548-4

**Published:** 2023-05-05

**Authors:** Yuwei Zhang, Ge Liao, Min Wang, Zhao Zhang, Liwei Liu, Yuqin Song, Dacheng Wang, Tingting Hao, Jie Feng, Bin Xia, Yixiang Wang, Xiaoyu Tang, Yihua Chen

**Affiliations:** 1 State Key Laboratory of Microbial Resources, Institute of Microbiology, Chinese Academy of Sciences, Beijing, 100101 China; 2 University of Chinese Academy of Sciences, Beijing, 100049 China; 3 Institute of Chemical Biology, Shenzhen Bay Laboratory, 518132 Shenzhen, China; 4 School of Biotechnology and Health Sciences, Wuyi University, Jiangmen, 529020 Guangdong China; 5 Renal Division, Peking University First Hospital, Peking University Institute of Nephrology, Beijing, 100034 China; 6 Department of Marine Pharmacy, College of Food and Pharmaceutical Sciences, Ningbo University, Ningbo, 315211 China; 7 Department of Pediatric Dentistry, Peking University School and Hospital of Stomatology, Beijing, 100081 China; 8 Central Laboratory Peking University School and Hospital of Stomatology, Beijing, 100081 China

**Keywords:** Tetramates, Mutanocyclin, Biosynthesis, Human microorganism, Adaptive evolution

## Abstract

**Background:**

Tetramates or tetramic acid-containing compounds (TACs) are a group of bioactive natural products featuring a pyrrolidine-2,4-dione ring acknowledged being closed via Dieckmann cyclization. The cariogenic *Streptococcus mutans* strains bearing a *muc* biosynthetic gene cluster (BGC) can synthesize mutanocyclin (MUC), a 3-acetylated TAC that can inhibit both leukocyte chemotaxis and filamentous development in *Candida albicans*. Some strains can also accumulate reutericyclins (RTCs), the intermediates of MUC biosynthesis with antibacterial activities. However, the formation mechanism of the pyrrolidine-2,4-dione ring of MUC and the distribution of *muc*-like BGCs along with their ecological functions has not been explored extensively.

**Results:**

We demonstrated that a key intermediate of MUC biosynthesis, M-307, is installed by a hybrid nonribosomal peptide synthetase-polyketide synthase assembly line and its pyrrolidine-2,4-dione ring is closed via an unprecedented lactam bond formation style. Subsequent C-3 acetylation will convert M-307 to RTCs, which is then hydrolyzed by a deacylase, MucF, to remove the N-1 fatty acyl appendage to generate MUC. Distribution analysis showed that the *muc*-like BGCs distribute predominantly in human-associated bacteria. Interestingly, most of the *muc*-like BGCs possessing a *mucF* gene were isolated from human or livestock directly, indicating their involvement in alleviating the host’s immune attacks by synthesizing MUC; while those BGCs lacking *mucF* gene distribute mainly in bacteria from fermented products, suggesting that they tend to synthesize RTCs to compete with neighboring bacteria. It is noteworthy that many bacteria in the same habitats (e.g., the oral cavity) lack the *muc*-like BGC, but possess functional MucF homologues to “detoxify” RTCs to MUC, including several competitive bacteria of *S. mutans*. We also comparably studied the distribution of TAS1, a fungal enzyme responsible for the production of phytotoxic tenuazonic acids (TeAs), a class of 3-acetylated TACs with similar structure but distinct biosynthetic mechanism to MUC, and found that it mainly exists in plants or crops.

**Conclusions:**

The in vivo and in vitro experiments revealed that the pyrrolidine-2,4-dione ring of MUC is closed via lactam bond formation, which may be adopted by many TACs without 3-acyl decorations. Besides, we found that *muc*-like BGCs are widespread in human-associated bacteria and their shapes and main products can be influenced by the habitat environment and vice versa. By comparing with TeAs, we provided thought-provoking insights into how ecological and evolutionary forces drive bacteria and fungi to construct a common 3-acetylated pyrrolidine-2,4-dione core through different routes, and how the biosynthetic processes are delicately controlled to generate diverse 3-acetylated TACs for environmental adaptation.

Video Abstract

**Supplementary Information:**

The online version contains supplementary material available at 10.1186/s40168-023-01548-4.

## Introduction

Tetramates or tetramic acid-containing compounds (TACs), featuring a pyrrolidine-2,4-dione ring, represent a large group of natural products from various terrestrial and marine organisms, including bacteria, fungi, and sponges [1–3]. TACs exhibit fascinating biological activities ranging from antibacterial, antifungal, antiviral, cytotoxic, to phytotoxic due to its significant structural diversities [2–5]. In recent years, hundreds of natural TACs have been isolated, while varied chemical synthesis strategies have been developed to expand the structural diversity of TACs [6, 7], and Spirotetramat (brand name Movento®), a synthetic TAC derivative possessing lipid biosynthesis inhibition activity, has been developed into an insecticide for crop protection [8].

**Fig. 1:**
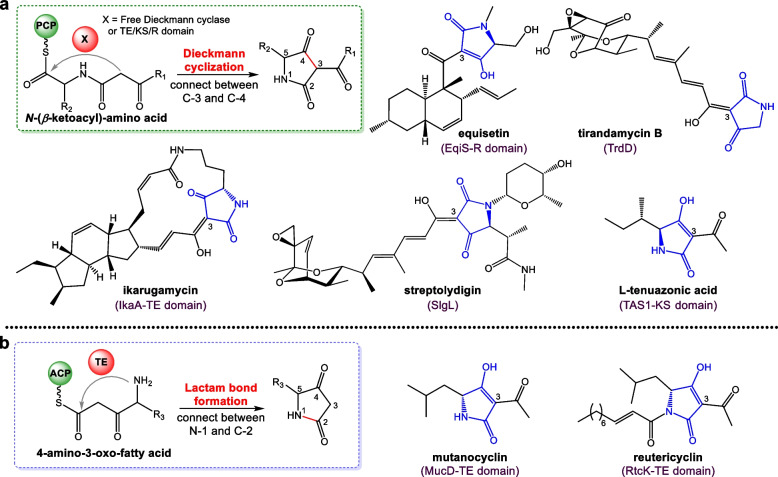
Two different mechanisms of the pyrrolidine-2,4-dione ring formation in TACs. **a** Representative TACs produced using Dieckmann cyclization mechanism. **b** The lactam bond formation mechanism proved in this study

In the past decade, investigations of TAC biosynthesis have revealed a universal Dieckmann cyclization scenario for the pyrrolidine-2,4-dione ring formation [9] (Fig. 1a). Most characterized TAC assembly lines are hybrid polyketide synthase (PKS)/non-ribosomal peptide synthetase (NRPS) enzymes with the *C*-terminal NRPS module containing at least three domains: a condensation (C) domain, an adenylation (A) domain, and a peptidyl carrier protein (PCP) domain. The NRPS C domain condenses the upper substrate, an acyl carrier protein (ACP) activated *β*-ketoacyl group, with a PCP-tethered amino acid to form a linear *N*-(*β*-ketoacyl)-amino acid intermediate, which is then offloaded and cyclized by a *C*-terminal reductase (R) [10], a thioesterase (TE) domain [11], or a free Dieckmann cyclase [12–14] via Dieckmann cyclization to give a pyrrolidine-2,4-dione ring (Fig. 1a, Additional file 1, Fig. S1).

Most TACs are acylated at the C-3 position of the pyrrolidine-2,4-dione ring, including some 3-acetylated TACs with a short C-3 acetyl modification. Pyrrolidine-2,4-dione ring installation in the structurally simple 3-acetylated TACs is not so “simple.” In addition to the general PKS/NRPS Dieckmann cyclization mechanism for 3-acetylated TACs, e.g., cyclopiazonic acid and Speradine A [15, 16] (Additional file 1, Fig. S1), NRPS/PKS assembly lines are proposed to be responsible for the biosynthesis of several 3-acetylated TACs, including Tenuazonic acids (TeAs), mutanocyclin (MUC), and reutericyclin (RTC) (Fig. 1), wherein the latter two were assumed to be installed through a non-Dieckmann mechanism [17, 18].

TeAs, represented by l-tenuazonic acid (l-TeA), are a group of 3-acetylated TACs isolated from *Alternaria* and some other phytopathogenic fungal species [19, 20]. They exhibit a broad spectrum of biological properties, including antitumor, antibacterial, and antiviral activities in addition to significant phytotoxicity [21, 22]. The biosynthesis of l-TeA has been well elucidated in the rice fungal pathogen *Pyricularia oryzae*. A hybrid NRPS/PKS enzyme TAS1 condenses the PCP-tethered l-isoleucine with an acetoacetyl-CoA to generate a PCP-tethered *N*-acetoacetyl-l-isoleucine intermediate, which is then offloaded and cyclized by the *C*-terminal KS domain to close the pyrrolidine-2,4-dione ring via a Dieckmann reaction [23] (Fig. 1a).

**Fig. 2:**
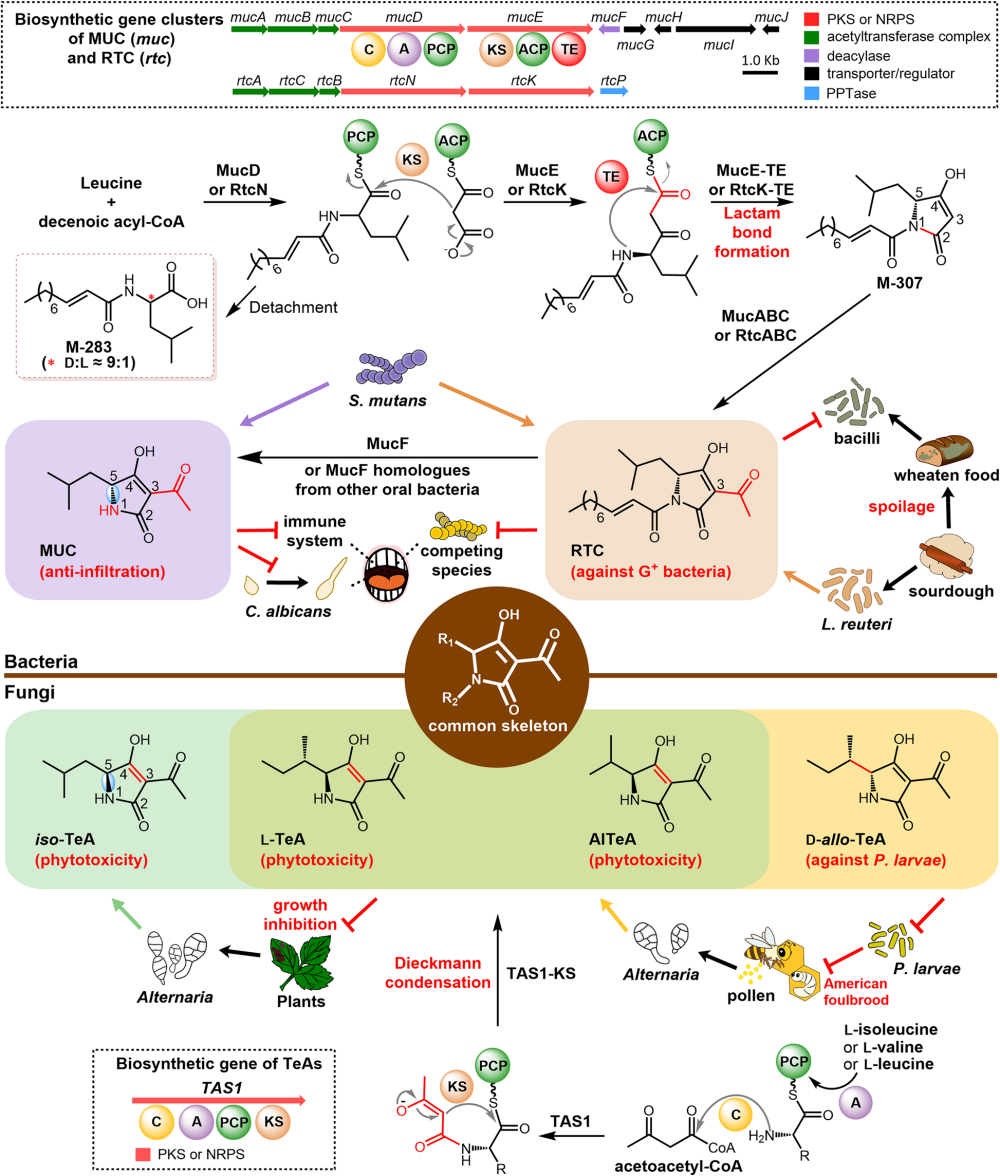
The different biosynthetic mechanisms of MUC and TeAs and their diverse ecological roles in hosts. MUC and RTC isolated from bacteria share a pyrrolidine-2,4-dione ring backbone with TeAs produced by fungi. MUC and RTC are generated through lactam bond formation differently from the prevalent Dieckmann cyclization mechanism as in TeAs. Strains containing the *muc* gene cluster, such as human oral bacteria *S. mutans*, can produce both the immunomodulator (MUC) and antibacterial agent (RTC), while the *rtc* gene cluster without *mucF* originally discovered in sourdough-generated *L. reuteri* can only produce RTC. Different *Alternaria* strains isolated from plants or insects can produce diverse TeAs with phytotoxic or antibacterial activities through the incorporation of different precursors

The anti-infiltration agent MUC from *Streptococcus mutans*, a human oral caries causative agent, is the C-5 (*R*)-configured enantiomer of *iso*-tenuazonic acid, one of the TeA isomers [24]. The proton-ionophore antibiotic RTC is the N-1 decenoic acylated MUC discovered in sourdough-isolated *Limosilactobacillus reuteri* strains [25]. Their biosynthetic gene clusters (BGCs, *muc* for MUC, and *rtc* for RTC) are shared with five conserved core genes [17]. Using *muc* BGC as an example, the core genes encode a NRPS/PKS assembly line (MucD/MucE) and three proteins (MucA/MucB/MucC) responsible for acetylation (Fig. 2). Moreover, RTC and its analogues with varied N-1 fatty acyl groups could be isolated from *S. mutans* B04Sm5 together with MUC, which was proposed to be generated by detaching the N-1 acyl group of RTCs by the deacylase, MucF [18]. Overall, previous studies have suggested that MUC and RTCs can be synthesized by the same NRPS/PKS machinery, and their biosynthetic pathways have been inferred [17, 18]. However, the formation mechanism of the pyrrolidine-2,4-dione ring in MUC and RTCs has not been proven yet.

The biosynthetic mechanisms and evolutionary relationships of those 3-acetylated TACs (MUC, RTCs, and TeAs) with similar structures but different biological properties attracted our attention for further investigation. The functions of MucD and MucE were dissected in this study, and we provided both in vivo and in vitro evidence showing that in addition to Dieckmann cyclization, the pyrrolidine-2,4-dione ring could be closed via lactam bond formation. Additionally, we found that many human-associated bacteria possess functional MucF homologues capable of “detoxifying” the antibacterial RTCs to MUC. The distributions and organizations of related BGCs and *mucF*-like genes were analyzed to understand this phenomenon. Generally, interesting insights into how bacteria and fungi evolve different mechanisms to install 3-acylated TAC skeletons and how they control the biosynthetic processes delicately to synthesize different compounds with diverse bioactivities to adapt to their habitats and influence their “neighbors” was provided.

## Results

### Production of MUC in *S. mutans* UA140

**Fig. 3:**
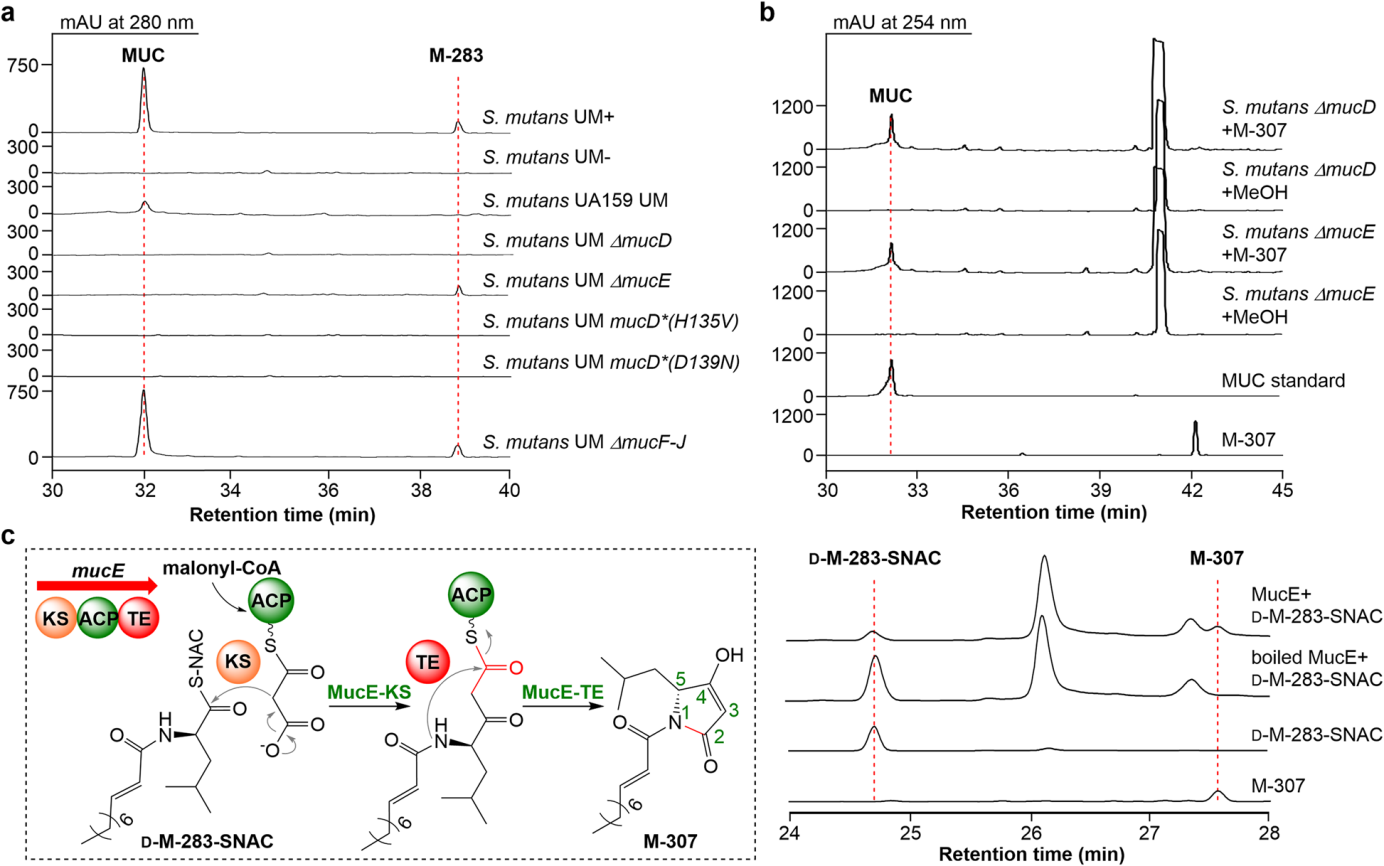
In vivo and in vitro characterization of MucD and MucE. **a** HPLC analysis of *muc* gene cluster heterologous expression strain *S. mutans* UM and the *muc* gene mutants. UM-, no xylose induction; UM + , xylose-induced. **b** HPLC analysis of *M-307* feeding experiments of *S. mutans* UM *ΔmucD* and *ΔmucE.* **c** HPLC analysis of the MucE assays using *d-M-283-SNAC* as a substrate

In the previous study, we successfully expressed the *muc* BGC from *S*. *mutans* 35 in a model strain *S*. *mutans* UA159 [24]. However, MUC production in *S. mutans* UA159 UM was quite low (Fig. 3a), making it difficult to study its biosynthesis. To overcome this issue, we employed *S. mutans* UA140 [26], another genetically amenable *S. mutans* strain, as a host for MUC production. The *muc* BGC was cloned into the same genome locus of *S. mutans* UA140 as the producer *S. mutans* 35 using the NabLC technique, and the *mucA-E* operon was put under the control of a xylose-inducible promoter *xylS1p* to generate *S. mutans* UM (Additional file 1, Fig. S2, S3). When the *S. mutans* UM cells were cultured in ASS (Artificial Saliva Substitutes) medium with 0.4% xylose, stable and high-level production of MUC was detected through HPLC (Fig. 3a). Therefore, this expression system was used in the following MUC biosynthesis studies.

### Characterization of *mucD*

The NRPS/PKS assembly line of MUC consists of two tri-domain proteins, including MucD (an NRPS with C-A-PCP domains) and MucE (a PKS with KS-ACP-TE domains). To study the role of *mucD*, we constructed its in-frame deletion mutant *S. mutans* UM *∆mucD* (Additional file 1, Fig. S4a, S5). Next, using multiple sequence alignment and phylogenetic analysis, the *N*-terminal C domain of MucD (MucD-C) was carefully analyzed, suggesting that it belongs to the starter C domain clade with conserved essential active site residues (Additional file 1, Fig. S6). Two *S. mutans* UM strains(mucD^*^(H135V) and mucD^*^(D139N)) with point mutations at their active site residues were constructed to verify the function of MucD (Additional file 1, Fig. S4b, S5). MUC production was blocked in all three mutants (Fig. 3a). Further careful inspection of the HPLC profiles revealed that a small peak at 39 min disappeared in those three mutants. Remarkably, inactivation of *mucE* did not influence the production of the peak at 39 min. According to this result, the peak represents an early offloaded intermediate from the NRPS assembly line. High resolution-mass spectrometry (HR-MS) of the compound (named M-283) resulted in an *m*/*z* of 284.2227 for [M + H]^+^ ion, matched well with a decenoic acyl-Leu structure (C_16_H_30_NO_2_, calcd 284.2220 for [M + H]^+^ ion) (Additional file 1, Fig. S7a). It was verified to be a mixture of (*E*)-dec-2-enoyl-d-leucine and (*E*)-dec-2-enoyl-l-leucine with a ratio of about 9:1 by comparing its (*S*)-1-(naphthalen-2-yl)ethan-1-amine derivatives with the chemically synthesized standards (Additional file 1, Fig. S7b). Previous precursor isotope labeling studies showed that only l-Leu could be incorporated into MUC [18]. Thus, the tri-domain NRPS, MucD, may catalyze the condensation of a decenoic acyl-CoA with an l-Leu and an epimerization.

### Characterization of *mucE*

To probe the function of MucE, a *mucE* in-frame deletion mutant (*S. mutans* UM *∆mucE*) was constructed (Additional file 1, Fig. S4c, S5), which lost the capacity to synthesize MUC but could still produce M-283 (Fig. 3a). Based on the structures of MUC and M-283, we proposed that MucE-KS condenses the MucD-PCP tethered M-283 and the MucE-ACP tethered malonyl group, after which the MucE-TE offloads the elongated linear chain and closes the pyrrolidine-2,4-dione ring through the lactam bond formation to give the deacylated RTC (named M-307). To test this hypothesis, M-307 was chemically synthesized and fed to *S. mutans* UM *∆mucD* or *∆mucE*. It resulted that both mutants could restore the production of MUC (Fig. 3b), indicating that M-307 is an intermediate formed by MucD and MucE.

Subsequently, we expressed MucE as an *N*-His_6_ tagged protein in *Escherichia coli* BAP1 containing the *sfp* gene, which encodes a phosphopantetheinyl transferase (PPTase) converting apo-MucE to its active holo-form post-translationally. Meanwhile, PKsC, a Malonyl-CoA: ACP transacylase (MAT) with high substrate flexibility toward the ACP domain of different trans-AT PKSs, was also expressed and purified for loading CoA-activated malonyl group onto MucE-ACP. The *N*-acetylcysteamine forms of (*E*)-dec-2-enoyl-d-leucine (d-M-283-SNAC) and (*E*)-dec-2-enoyl-l-leucine (l-M-283-SNAC) were synthesized to mimic their PCP tethered state. Then, the catalytic activity of Holo-MucE was tested in assays with PKsC, malonyl-CoA, and d-M-283-SNAC or l-M-283-SNAC. M-307 was generated only in the case of d-M-283-SNAC (Fig. 3c, Additional file 1, Fig. S8), suggesting that MucE can catalyze the Claisen condensation between d-M-283-SNAC and the ACP-tethered malonyl group, release the elongated chain, and close the pyrrolidine-2,4-dione ring via lactam bond formation.

Both mimic substrate d-Leu-SNAC and putative product M-155 were synthesized to check whether MucE could accept PCP-tethered d-Leu. The incubation of d-Leu-SNAC with holo-MucE, PKsC, and malonyl-CoA failed to generate M-155 or any other product (Additional file 1, Fig. S9), verifying that the N-1 fatty acyl initiation catalyzed by MucD-C is necessary for MUC assembly.

### Genes *mucF–J* are not necessary for MUC biosynthesis in *S. mutans* UM

**Fig. 4:**
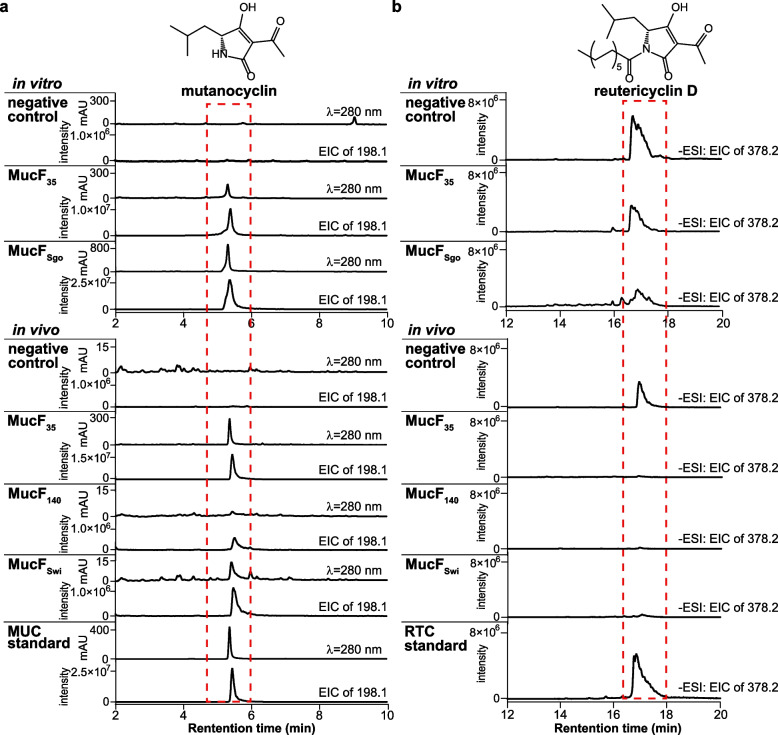
Characterization of MucF and its homologous proteins as deacylase. **a** LC–MS analysis of MucF and its homologous proteins in converting RTC to MUC. MUC was detected. **b** LC–MS analysis of MucF and its homologous proteins in converting RTC to MUC. RTC was detected. The cell lysate of *E. coli* Rosetta 2(DE3)pLysS/pET28a supplemented with RTC was used as the negative control for in vitro assays; *E. coli* BAP1::*mucA-E* (*E. coli* BAP1 containing pEXT06), which can produce RTC and does not contain MucF, was used as another negative control for in vivo assays. The function of MucF_35_ was verified both in vitro and in vivo; MucF_140_ represents MucF homologous proteins from non-MUC-producing *S. mutans* strains; MucF_Swi_ and MucF_Sgo_ were derived from *Scardovia wiggsiae* and *S. gordonii* respectively, representing MucF homologous proteins from oral bacteria other than *S. mutans*. See the LC–MS results of other MucF homologues in Additional file 1, Fig. S11, S12

It was proposed that M-307 can be modified by MucABC to add a C-3 acetyl group to generate RTC, which is then converted to MUC by an unusual peptidase encoded by *mucF*. Unexpectedly, the production of MUC in *S. mutans* UM ∆*mucF*–*J* (Fig. 3a), a mutant with the *mucF*–*J* gene cassette being deleted (Additional file 1, Fig. S4d, S5), was not influenced. This suggests that the five genes are not essential for the production of MUC in *S. mutans* UM. MucG and MucH are TetR/AcrR family transcriptional regulators, MucI is a DHA2 family efflux MFS transporter permease subunit [27], and MucJ is a putative multi-drug export protein (Additional file 1, Table S1). Since the *mucA*–*E* operon was under the control of a xylose-inducible promoter in *S. mutans* UM, it was not surprising that the regulatory genes were unnecessary. The functions of MucI and MucJ may be compensated by other exporters. However, the peptidase activity of MucF should be essential for MUC biosynthesis. To probe the function of *mucF*_*35*_ from *S. mutans* 35, we first cloned it into pET28a and expressed this membrane protein in *E. coli*. MUC production could be detected when the cell lysate was incubated with RTC (Fig. 4), verifying the RTC deacylase activity of MucF_35_. Additionally, we conducted an in vivo assay in which the codon-optimized *mucF*_*35*_ was inserted into pEXT06 (containing *mucA*–*E* for synthesizing RTCs in *E. coli*), and the *E. coli* strain harboring this plasmid gained the ability to produce MUC as anticipated (Fig. 4a). These results implied that there are other proteins in *S. mutans* UM ∆*mucF*–*J* capable of rescuing the loss of MucF. A BLASTP search of the *S. mutans* UA140 genome for MucF-like proteins resulted in a homologous protein, MucF_140_ that shares 55.9% identity with MucF_35_. When MucF_140_ was characterized in vivo using the same assay as that for MucF_35_, it could act as an efficient RTC deacylase (Fig. 4), which explains why *S. mutans* UM ∆*mucF*–*J* can still synthesize MUC.

### Distribution of the *muc*-like BGCs and the *mucF*-like genes

Some *S. mutans* with the *muc* BGC, like the strain B04Sm5, can synthesize both RTCs and MUC. RTCs are antibiotics that inhibit the growth of many Gram-positive bacteria, including the competitors of *S. mutans* in the oral cavity [18], while MUC acts as an immunomodulator to protect bacteria from the host’s immune attack and a regulator of filamentous development in *Candida albicans* [28], an opportunistic fungal pathogen. The fact that *S. mutans* UA140, which does not produce RTCs, has a MucF-like peptidase capable of hydrolyzing RTCs to MUC implies that some bacteria can take advantage of the chemical weapons produced by bacteria harboring the *muc* or *rtc* BGC, which aroused our interests to determine those “smart” bacteria by distribution investigation of the *muc*-like BGCs and the *mucF*-like genes.

**Fig. 5:**
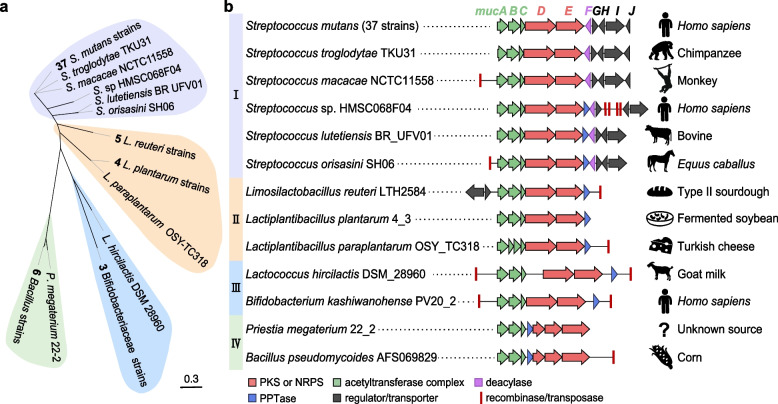
Phylogeny, organization, and distribution of the *muc*-like BGCs. **a** The Maximum likelihood tree of nucleotide sequences covering *mucA*-*E* from the 63 *muc*-like BGCs. Strains belonging to the same species were plotted as one branch; see the detailed tree showing information of all strains in Additional file 1, Fig. S9. The four types of *muc*-like BGCs were represented by different colors. **b** Organization and distribution of the four types of *muc*-like BGCs. The hosts of strains are indicated by black icons and text on the right. Transposases or recombinases at the adjacent position are marked with red bars

Distribution analysis of the *muc*-like BGCs was conducted by searching the core genes of *muc* BGC, including *mucA-E*, which resulted in 63 strains belonging to Firmicutes (60 strains) and Actinobacteria (3 strains) (Additional file 1, Table S2). Strains of the former phylum include 37 *S. mutans* (14% of the 264 sequenced strains) and 23 other Bacilli; strains of the latter phylum include two *Bifidobacterium*, and one strain belonging to Bifidobacteriaceae Family. Significantly, most of the 63 strains were isolated from the human body (40 strains) or human-related environments (18 strains), such as fermented products, livestock, and crops. Further phylogenetic analysis of the nucleotide sequences spanning *mucA*-*E* could group those BGCs into four types (Fig. 5, Additional file 1, Fig. S10). Type I BGCs are distributed exclusively in the genus *Streptococcus* (42 strains) of which 38 strains are from the human body, including the *S. mutans* from the oral cavity (32 strains), ICU (2 strains), infective endocarditis tissues (2 strains), duodenal aspirate (1 strain), and the *S.* sp. HMSC068F04 strain from sputum. Additionally, four *Streptococcus* strains were either isolated from the livestock or the oral cavity of primates. The type I BGCs all share the same genetic organization as the *muc* BGC (Fig. 5b), and the presence of the *mucF* homologues suggests MUC could be the main product. Type II BGCs can be found in *Limosilactobacillus* and *Lactiplantibacillus* genus (10 strains, Additional file 1, Table S2), with eight strains isolated from fermented foods or animal feed, including four *L. reuteri* strains from industrial sourdough continuously used in the same factory (Fig. 5b). The other two Lactobacillaceae strains were isolated from *Odontesthes bonariensis* and Lion-tailed macaque. Most type II BGCs lack the *mucF*-like gene. This suggests that their products are RTCs retaining the N-1 fatty acyl decoration. Type III BGCs were observed in the *Lactococcus hircilactis* DSM 28,960 from goat milk and three Bifidobacteriaceae strains from human and fermented food. They have some additional insertions inside the *mucA-E* and the PPTase gene operon. The seven *muc*-like BGCs from the *Bacillus* and *Priestia* strains comprised Type IV BGCs, where the homologous gene of *mucD* is split into two genes. Apart from *Bacillus cereus* IZSPB_BC561A (isolated from ice cream) and *Priestia megaterium* 22–2 (unknown source), the other five *Bacillus* strains were obtained from plants. Type III and IV BGCs lack the *mucF*-like gene and may also synthesize RTCs. Notably, except for the clusters from *S. mutans*, transposase or recombinase encoding genes could be frequently observed in the vicinity of the *muc*-like BGCs. This implies the involvement of horizontal gene transfers (HGTs) to promote their spreading.

**Fig. 6:**
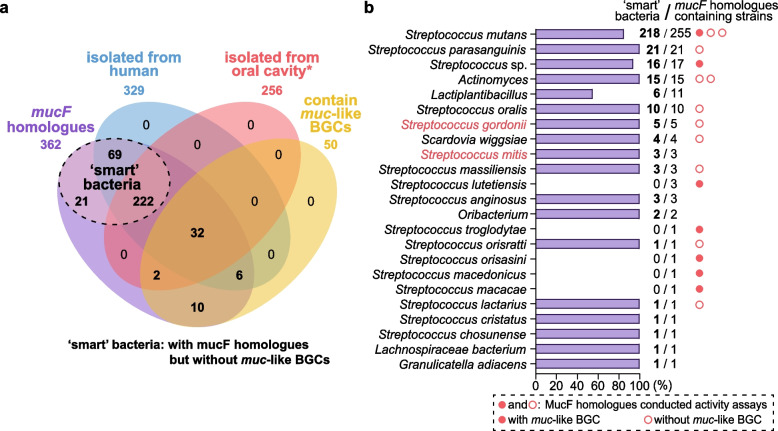
Distribution of the MucF homologues characterized in this study. **a** The Venn diagram shows the number of *mucF* homologues containing strains from the human body (blue shade) and the oral cavity (red shade) or with a *muc*-like BGC (yellow shade). Strains with *mucF* homologues but without the *muc*-like BGCs were considered as “smart” bacteria. Oral cavity* represents the oral cavity of humans and two primate animals. **b** The diagram shows the proportion and number of “smart” bacteria in different species, with some known competitors of *S. mutans* marked in red font. MucF_35_ and the other 17 MucF homologous tested for deacylase activity in this work were marked with red dots (from strains containing a *muc*-like BGC) or circles (from strains without *muc*-like BGC). Details of each protein are described in Additional file 1, Table S3

A subsequent search using MucF_35_ as a probe resulted in 111 MucF-like proteins existing in 362 strains (some *mucF*-like genes from different strains dictate the same protein) (Fig. 6a, Additional file 1, Table S3). Significantly, most hits (312 strains) do not have the other genes for MUC biosynthesis, and some genera, such as *Actinomyces*, *Granulicatella*, *Lachnospira*, *Oribacterium*, and *Scardovia*, have never been reported to produce either MUC or RTCs. Even for *S. mutans*, the main producer of MUC and RTCs, only 37 of the 255 strains with a *mucF* homologue possess *muc* BGC, the other 218 strains may share competitive advantages by only expressing the MucF-like proteins. Further habitat analysis of those strains revealed that about 90% of them (329 strains) are from the human body and more than 70% (254 strains) are directly from the oral cavity. Interestingly, a small number of the *Streptococcus* competitors of *S. mutans* in the oral cavity, e.g., *Streptococcus gordonii* (3 of 85 strains) and *Streptococcus mitis* (2 of 174 strains), have also acquired the *mucF* homologues for defense.

Sixteen of the MucF-like proteins were selected, synthesized, and tested using the same assays as those for MucF_35_, including six from non-*mutans Streptococcus* strains with the *muc* BGC and 10 from strains without the cluster to verify their functions (Fig. 6b, Additional file 1, Fig. S11, S12, Table S3). In the latter group, we specifically chose two MucF-like proteins from *Actinomyces* isolated from human dental plaque and liver abscess, respectively, one from *Scardovia wiggsiae*, a causative agent for early childhood dental caries, and one from *S. gordonii*, a known competitor of *S. mutans*. Results showed that all 16 MucF homologues could hydrolyze RTC into MUC regardless of whether they were from strains with or without the *muc* BGC, strongly supporting a wide distribution of the “smart” bacteria that have gained the ability to survive under the stress of RTCs in certain human-related environments.

## Discussion

Bacterial origin MUC and RTCs are 3-acetylated TACs structurally similar to TeAs from fungi. As aforementioned, the pyrrolidine-2,4-dione core of TeAs and many other TACs are formed via Dieckmann cyclization. It was shown that MucD starts MUC biosynthesis by establishing M-283 whose d-isomer is further elongated and cyclized by MucE to form the pyrrolidine-2,4-dione moiety via a lactam bond formation mechanism to generate M-307 in this study. A clear difference between TACs synthesized via these two ways is that the pyrrolidine-2,4-dione moiety installed via Dieckmann cyclization bears an innate 3-acyl chain from its *β*-ketoacyl precursor. In contrast, those installed via lactam bond formation have no C-3 decoration unless added by tailoring enzymes (Fig. 2). Many naturally occurring TACs do not actually possess C-3 acyl appendage. They either have no C-3 decoration (e.g., belamide A [29], eliamid [30], or sintokamides [31]) or possess a simple C-3 methyl group (e.g., palauimide [32] and tetrapetalones [33]) (Additional file 1, Fig. S13). Further biosynthetic investigations of those natural products may reveal more TACs constructed via lactam bond formation. It was proposed that MucABC further acetylates M-307 to generate RTCs, which are finally converted to MUC by the MucF-catalyzed N-1 deacylation for MUC biosynthesis.

As mentioned above, RTCs are ionophore antibiotics capable of inhibiting the growth of many Gram-positive bacteria; MUC exhibits no antibiotic activity but can inhibit leukocyte chemotaxis and relieve the immune pressure from the host [22]. It can also suppress the hyphal formation and virulence of *C. albicans* [28], a familiar commensal existing in many body sites, such as the human oral cavity. The analysis of the 63 strains containing *muc*-like BGCs strongly supported that ecological adaptation is a driving force shaping the BGCs and their products. All type I BGCs with *mucF* are from *Streptococcus* strains dwelling in humans, primates, and livestock, which face the host immune pressure and drastic competition from neighboring microbes. Since zoonotic transmission of microbes including *Staphylococcus aureus*, *Campylobacter* spp., and *L. reuteri* has been documented [34–36], we cannot preclude the possibility that these *muc*-like BGCs containing strains isolated in primates and livestock may have been transmitted from humans. Strains harboring type II–IV BGCs are mainly isolated from fermented products, crops, and plants. Similarly, since some strains of *L. reuteri* and *Lactiplantibacillus plantarum* can permanently or transiently colonize the intestinal tract of humans and animals, they may also have the opportunity to obtain *muc*-like BGCs through HGT from bacteria like *Streptococcus* strains dwelling in humans. The missing of *mucF* in most BGCs of these strains may suggest that they no longer need to deal with immune pressure and, therefore, mainly synthesize the antibacterial product RTCs. Notably, some *S. mutans* strains can produce both RTCs (to inhibit the growth of a plethora of oral microbes, including *Rothia mucilaginosa* and some *Streptococcus* strains) and MUC (to modulate the host environment) [18], which may be achieved through the delicate control of the expression and/or catalytic activity of MucF. This phenomenon indicates that even within *S. mutans* strains containing the *muc* BGCs, there are differences in their ability to produce RTC/MUC. One limitation of this study is that we have not verified the function of *muc*-like BGCs in mixed communities. Exploring the impact of *muc* BGC-containing strains on colonization and abundance of *S. mutans* populations through in vitro oral biofilm or animal models will help us further understand the ecological role of *muc* BGCs.

Considering that about 2/3 of the 63 strains are human-associated bacteria, and most of the others are from fermented food, livestock, and crops, it was proposed that human beings play an essential role in spreading the *muc*-like BGCs. Such HGT events from human commensals (e.g., *S. mutans*) to closely related bacteria in human-related environments are supported by the presence of transposase or recombinase genes flanking the BGCs from strains other than *S. mutans*. After that, selection pressures from habitats of the receptor strains will reshape the BGCs, guiding them to produce MUC or RTCs. The four RTCs producing *L. reuteri* strains repeatedly isolated from industry sourdough of the same bakery may be the result of such an adaptation process (Fig. 2). It was reported that the bread dough prepared with 6–24% sourdough fermented with the RTCs producing *L. reuteri* strain could inhibit the bread spoilage caused by *Bacillus* and extend shelf-life [37]. Since *Bacillus* spp. can form heat-resistant endospores that are difficult to destroy while baking [38, 39], the thermal stable RTCs further highlight their beneficial effects. Therefore, the Lactobacillaceae strains lacking the *mucF* gene tend to be artificially selected for high-quality fermented food preparation. Such HGT events between *S. mutans* and Lactobacillales from human-related environments are not rare. *S. mutans* UA140 and *Lactococcus lactis* KF147 (from mung bean sprouts) share a similar TnSmu2 gene cluster capable of improving the host’s oxidative stress resistance capacity [40]. Research on the homologous BGCs shared by human commensals and microbes from human living environments may uncover more cases of similar adaptive evolution.

An interesting phenomenon is the widespread distribution of *mucF*-like genes in human-associated microorganisms. They dwell mainly in the same habitats as the bacteria containing a *muc*-like BGC, especially in the oral cavity. Moreover, all 11 tested MucF homologues from strains without the *muc*-like BGC worked well as RTC deacylases, indicating that those “smart” bacteria can benefit from the neighbor-made chemical weapons by hydrolyzing the toxic RTCs to protective MUC. Notably, among the 264 genome-sequenced *S. mutans* strains, 255 possessed a *mucF* homologue of which 37 contain the *muc* BGC and 218 do not, which may help the *S. mutans* species outcompete the oral flora and promote dental caries development. The *mucF* homologue can also be found in *S. wiggsiae*, which is another caries-associated agent that can exist in teeth plaque together with *S. mutans* [41, 42], implying a potential synergistic relationship between the two species. Meanwhile, a small group of the competitive bacteria of *S. mutans*, like several *S. gordonii* and *S. mitis* strains, also acquire the *mucF*-like gene during the long co-existence and combat with *S. mutans* in the oral cavity. Generally, the wide spread of *mucF* homologues in oral bacteria reveals how the *muc* BGC, a result of adaptive evolution in several *S. mutans* strains, further influences their “neighbors” in the same community, whether they are “friends” or “foes.” A similar phenomenon was observed in a recent work about the ecological role of colibactin [43], which can induce DNA damage and prophage induction. The *clbs* gene in its BGC encodes a cyclopropane hydrolase and plays a protective role. It was revealed that *clbs* homologues can be found in and protect non-colibactin-producing *E. coli* strains along with members of the human and honey bee gut microbiome.

TeAs from fungi are very similar to MUC in structure. Specifically, *iso*-TeA (5*S*) and MUC (5*R*) are a pair of enantiomers although they are biosynthesized through totally different routes. It was found that the TeA producers, e.g., *Alternaria brassicicola* and *Alternaria raphani*, could produce d-*allo*-TeA with 5*R* configuration as a minor product [44]. This implies that some fungi can synthesize MUC, which raises the question: why do fungi produce TeAs instead of MUC? To understand the impact of habitat environment on the stereochemical preference of TAS1, we analyzed the distribution of TeA synthetase using the TAS1 from *P. oryzae* 70–15 as a probe, which resulted in 97 retrievals spreading in two fungus phyla, Basidiomycota (12) and Ascomycota (85) (Additional file 1, Table S4). Considering that the phytotoxicity of TeAs may help fungi colonize and infect plants, it is not surprising that many *TAS1-*containing fungi, including many phytopathogenic fungal species like *Aspergillus*, *Pyricularia*, and *Fusarium*, are from plants or crops worldwide. Additionally, ten *TAS1-*containing fungi were from insects (e.g., *Popillia japonica*, *Spodoptera litura*, and *Locusta migratoria manilensis*) and/or their nests. Specifically, it was shown that l-TeA, AITeA, and d-*allo*-TeA produced by *Alternaria* strains from beehive pollens could protect bee brood from American foulbrood by inhibiting the pathogenic bacterium *Paenibacillus larvae* [44]. However, no *TAS1-*containing fungus has been isolated from a niche facing the immune pressure from vertebrates, although this result may be constrained by the number of fungi isolated and sequenced in vertebrates currently. MUC and TeAs with similar structures present a good example of how bacteria and fungi adopt different biosynthetic routes to generate enantiomers with distinct bioactivities to help the producers adapt to their habitats.

Conclusively, we showed that MUC closes the pyrrolidine-2,4-dione ring via lactam bond formation and proposed that such a non-Dieckmann cyclization mechanism may be adopted by many TACs without 3-acyl decoration. Comparisons of the producers, biological roles, and biosynthetic mechanisms of MUC, RTCs, and TeAs are summarized in Fig. 2. The figure depicts an interesting case of convergent evolution, in which ecological and evolutionary forces drive bacteria and fungi to assemble a common 3-acetylated pyrrolidine-2,4-dione core through totally different manners. In addition, it shows how fungi and bacteria can diversify the structures and activities of natural products with this general skeleton to adapt to environments and even influence the shape of their “neighbors.” It unveils a corner of the everlasting evolution and screening process of life and may encourage the use of 3-acetylated TACs to improve oral health and inspire the synthetic biology efforts to further expand the chemical space of TACs.

## Conclusions

In this study, we demonstrate that the *muc* BGC produces mutanocyclin (MUC), an immunomodulator, and reutericyclins (RTCs), the antibacterial agent, through lactam bond formation. Interestingly, this differs from the usual Dieckmann cyclization observed in phytotoxic TeAs produced by TAS1, despite these compounds sharing the pyrrolidine-2,4-dione ring backbone. The *muc-*like BGCs mainly exist in human-related bacteria, with many of their “neighbors” acquiring MucF homologues detoxifying RTCs, while TAS1 mainly exists in fungi from plants or corps. Strains originating from diverse habitats produce 3-acetylated TACs through distinct biosynthetic routes, resulting in varying activities. These findings provide potential evidence of how ecological and evolutionary forces shape the behavior of microorganisms.

## Materials and methods

### Bacterial strains, plasmids and culture conditions

Bacterial strains and plasmids used in this study are listed in Additional file 1, Table S5. *S. mutans* strains were grown anaerobically on BHI agar plates or in BHI broth (BHI; OXOID LTD., Basingstoke, England) at 37 °C. The modified ASS (artificial saliva substitutes) medium was prepared as described [22] and used specifically for basic fermentation and feeding experiments. BHI plates containing 4 mg/mL *p*-Cl-Phe were used for the selection of *p*-Cl-Phe-sensitive colonies. For the selection of antibiotic-resistant colonies, BHI plates were supplemented with erythromycin (12.5 μg/mL) and LB plates were supplemented with chloramphenicol (27 μg/mL) and kanamycin (50 μg/mL).

### General DNA manipulations

All PCR primers used in this study were synthesized in Genery Co., Ltd. (Shanghai, China) and listed in Additional file 1, Table S6. Common DNA sequencing and genome sequencing of *S. mutans* B30 was performed by Novogene (Beijing, China). The genes encoding different MucF homologous proteins were codon optimized and synthesized by Tsingke Biotechnology Co., Ltd. (Beijing, China). PCR reactions were carried out with Taq DNA polymerase (TransGene, Beijing, China) or Phusion® HF DNA polymerase (Thermo Fisher scientific, MA) according to the manufacturers’ instructions. Golden Gate cloning and overlapping PCR were performed following the literature procedures [45]. Preparation of genomic DNAs of different *S. mutans* strains was performed as described [22]. For natural transformation experiments, cells were maintained in Todd-Hewitt medium (Becton–Dickinson, Sparks, MD) supplemented with 0.3% yeast extract. A detailed natural transformation procedure was described previously [46].

### Heterologous expression of the *muc* gene cluster in *S. mutans* UA140

The *muc* gene cluster (*mucA*–*J*) locates at a genomic island in *S. mutans* 35, and sequences flanking this genomic island are well conserved in *S. mutans* UA140. Therefore, the *muc* gene cluster was cloned to the same genome locus of *S. mutans* UA140 using the flanking sequences as homologous arms via the NabLC technique (Additional file 1, Fig. S2). To construct the recipient strain, the 2.1-kb IFDC2 cassette [47] that contains the erythromycin resistance gene (*ermAM*) and the counter-selective marker gene (pheS^*^) was PCR cloned with primer pair ldh-F/erm-R using pIFDC2 as a template. The 0.6-kb left capture arm (CAL) and the 0.6-kb right capture arm (CAR) were PCR amplified from *S. mutans* UA140 genomic DNA with primer pairs UA140-upF/UA140-upR-erm and UA140-dnF-ldh/UA140-dnR, respectively. The three fragments were mixed as the template for an overlapping PCR with primer pair UA140-upF/UA140-dnR to generate a 3.3-kb fragment CAL-IFDC2-CAR, which was then transformed to *S. mutans* UA140. Colonies resistant to erythromycin on BHI plates were selected and PCR-verified with primers UA140-upF1/UA140-dnR1 as the recipient strain *S. mutans* UA140-RS/MUC. Genomic DNAs of *S. mutans* 35 were then transformed to *S. mutans* UA140-RS/MUC via natural transformation, and the colonies that could grow on BHI plates containing 4 mg/mL *p*-Cl-Phe were selected as positive transformants. The positive transformants that could not grow on BHI plates with erythromycin were picked and PCR-verified using primers UA140-upF1/orfJ, bacA-F/bacA-R, orfE-F/orfE-R, and orfA/UA140-dnR1 as the *S. mutans* UM^*^ (Additional file 1, Fig. S2).

To insert the xylose-inducible promoter *xylS1*_*P*_, the 1.4-kb fragment containing the *xylS1*_*P*_ promoter was amplified from pZX9 [48] using primer pair XylRR-BsaI/XylOR-BsaI. The 2.1-kb IFDC2 cassette was amplified using pIFDC2 as a template with primer pair ermR-BsaI/ldhF-BsaI. Two 0.7-kb and 0.6-kb fragments flanking the insertion site were PCR cloned with primers orfA-upF/orfA-upR2-BsaI and orfA-dnF2-BsaI/orfA-dnR. The four fragments were ligated by Golden Gate cloning and transformed into *S. mutans* UM^*^. Colonies growing on BHI with erythromycin were selected and PCR-verified as *S. mutans* UM^*^/IFDC2-*xylS1*_*P*_ with primers orfA-upF/orfA-dnR. The IFDC2 cassette was then removed by counterselection via homologous recombination. The 0.7-kb and 2.0-kb fragments flanking the IFDC2 cassette were PCR cloned with primers orfA-upF/orfA-upR3-BsaI and XylRR1-BsaI/orfA-dnR using *S. mutans* UM^*^/IFDC2-*xylS1*_*P*_ genomic DNAs as a template. The two amplicons were ligated using Golden Gate cloning and transformed into *S. mutans* UM^*^/IFDC2-*xylS1*_*P*_. Colonies that were resistant to BHI plates containing 4 mg/mL *p*-Cl-Phe were selected and PCR-verified using primers orfA-upF/orfA-dnR as *S. mutans* UM (Additional file 1, Fig. S3).

### Construction of the *muc* gene in-frame deletion or point mutated strains

Construction of the *mucD* gene in-frame deletion mutant (*S. mutans* UM *∆mucD*) was completed via two rounds of homologous recombination (Additional file 1, Fig. S4a). The 0.8-kb fragment and 0.9-kb fragment flanking the *mucD* gene was PCR amplified with primers DUF/DUR and DDF/DDR using the genomic DNA of *S. mutans* UM as the template. The 2.1-kb IFDC2 cassette was PCR amplified with primer pair ldhF-BsaI/ermR-BsaI using pIFDC2 as a template. The two fragments flanking *mucD* and the IFDC2 cassette were ligated via Golden Gate cloning and transformed to *S. mutans* UM. Colonies resistant to erythromycin on BHI plates were selected and PCR-verified with primer pair DSupF/DSdnR as the *mucD* disrupted mutant, in which *mucD* was replaced with the IFDC2 cassette. The elimination of the IFDC2 cassette was achieved via the second round of homologous recombination. Briefly, the 0.8-kb fragment and 0.9-kb fragment flanking the IFDC2 cassette were PCR amplified with primers DUF/DUR and DDF2/DDR, ligated via Golden Gate cloning, and transformed to the *mucD* disrupted mutant. Colonies that could grow on BHI plates supplemented with *p*-Cl-Phe were selected, PCR-verified as *S. mutans* UM *∆mucD* with primer pair DSupF/DSdnR (Additional file 1, Fig. S5), and confirmed by sequencing.

In vivo point mutagenesis of the C-domain of MucD was carried out using the same strategy as that of *S. mutans* UM *∆mucD* to generate *S. mutans* UM mucD*(H135V) and mucD*(D139N), and different point mutations were introduced by the PCR primers at the second round of homologous recombination (Additional file 1, Fig. S4b). The 0.8-kb fragment and 1.0-kb fragment flanking *mucD-C* were PCR amplified with primer pairs CdHIUF/CdHIUR and CdHIDF/CdHIDR (to generate H135V mutation) and CdDIUF/CdDIUR and CdDIDF/ CdDIDR (to generate D139N mutation). The 2.1-kb IFDC2 cassette was amplified with primer pair ldhF-BsaI/ermR-BsaI. The three fragments were ligated and used for the first round of homologous recombination. For the removal of IFDC2 and the introduction of C domain point mutation, two 0.8-kb and 1.0-kb fragments were amplified with primer pairs CdHIUF/CdHUR and CdHDF/CdHIDR (to generate H135V mutation) and CdDIUF/CdDUR and CdDDF/CdDIDR (to generate D139N mutation). The MucD-C domain point mutations were PCR verified using primer pair CdmF/CdmR and confirmed by sequencing (Additional file 1, Fig. S5).

*S. mutans* UM* ∆mucE* was constructed in a similar manner, except that overlapping PCR technique, instead of Golden Gate cloning, was used in ligation of the two homologous fragments with the IFDC2 cassette (Additional file 1, Fig. S4c). The 0.8-kb and 0.6-kb fragments flanking *mucE* were amplified using primer pairs EUF/EUR and EDF/EDR, respectively. The 2.1-kb IFDC2 cassette was PCR amplified with primer pair ldhF/ermR. The *mucE* disrupted mutant was PCR verified using primer pair ESupF/ESdnR. For the IFDC2 cassette elimination, the 0.8-kb left homologous arm was amplified using primer pair EUF/EUR2, and the 0.6-kb right arm was amplified using primer pair EDF2/EDR. The successful construction of *S. mutans* UM *∆mucE* was PCR verified with primer pair ESupF/ESdnR and confirmed by sequencing (Additional file 1, Fig. S5).

The *mucF*–*J* locus in-frame deletion mutant *S. mutans* UM ∆*mucF*–*J* was constructed via two rounds of homologous recombination similar to the constructions of *S. mutans* UM *∆mucE* (Additional file 1, Fig. S4d). The 0.8-kb and 0.7-kb fragments flanking the *mucF*–*J* locus were PCR amplified with primer pairs JUF/JUR and FDF/FDR, respectively. The 2.1-kb IFDC2 cassette for counterselection was amplified using primer pair ldhF-MucJ/ermR1-MucF. The two fragments and the IFDC2 cassette were assembled by overlapping PCR to generate a 3.6-kb fragment for the construction of the *mucF*–*J* disrupted mutant, which was PCR-verified with primer pair MucJ-upF/MucF-dnR. For the IFDC2 cassette elimination, the 0.8-kb and 0.7-kb fragments were PCR amplified with primer pairs JUF/JUR2 and FDF2/FDR, ligated via overlapping PCR, and transformed to replace the IFDC2 cassette to generate *S. mutans* UM ∆*mucF*–*J*, which was PCR verified with primer pair MucJ-upF/MucF-dnR and confirmed by sequencing (Additional file 1, Fig. S5).

### Production of MUC

*S. mutans* UM and all the mutants were inoculated into 100 mL ASS medium with 0.4% xylose and cultured statically at 37 °C for 48 h. The cell pellet was discarded after centrifugation (5000 × *g*, 30 min) and the supernatant was extracted twice with 100 mL EtOAc. The combined organic layers were evaporated *in vacuum* and re-dissolved in 1 mL methanol for HPLC detection. HPLC detection of MUC was carried out on a Shimadzu HPLC system (Shimadzu, Kyoto, Japan) using a C18 column (4.6 × 250 mm, 5 μm, Apollo, Alltech, Lexington, Kentucky, USA) developed with solvent A (H_2_O with 0.1% (v/v) formic acid) and B (acetonitrile) at a flow rate of 1.0 mL/min. The percentage of acetonitrile was kept at 5% over 0–5 min, changed from 5 to 40% over 5–25 min, from 40 to 100% over 25–40 min, and kept at 100% over 40–45 min.

For the feeding experiments, *S. mutans* UM *∆mucD* or *∆mucE* was inoculated into 100 mL ASS medium with 0.4% xylose and M-307 (0.03 mM). After being cultured statically at 37 °C for 48 h, the supernatant was treated and analyzed under the same conditions as the fermentation broth of *S. mutans* UM.

### Expression and purification of MucE and PKsC

The 3.2-kb *mucE* gene was amplified as three fragments with primer pairs T7F/mucER1, mucEF2/mucER2, and mucEF3/T7R and assembled via overlapping PCR. After sequencing verification, the fragment was digested with *Nde*I/*Xho*I and inserted into the same sites of pET28a to generate pET28a-MucE, which was then transformed into *E. coli* BAP1 to generate *E. coli* BAP1/pET28a-MucE. A single transformant was inoculated into LB with 50 μg/mL kanamycin and cultured overnight at 37 °C, 220 rpm. The overnight seed was used to inoculate the same medium at 1:100 dilution and incubated at 37 °C, 220 rpm until OD_600_ reached 0.6. After the addition of 0.1 mM isopropyl-*β*-thiogalactoside (IPTG), the cells were further cultured at 16 °C, 180 rpm for 16–18 h. Purification of *N*-His_6_ tagged MucE was performed with the Ni–NTA affinity column at 4 °C following the manufacturer’s instructions.

Similarly, the 0.9-kb fragment containing the gene encoding PKsC was PCR cloned with primer pair PKsCF/PKsCR from *Bacillus subtilis* sp. 168 and verified by sequencing. The plasmid pET28a-PKsC was constructed by digesting the fragment with *Bam*HI/*Eco*RI and inserting it into pET28a. Then the plasmid was transformed into *E. coli* BL21 (DE3) to afford *E. coli* BL21 (DE3)/pET28a-PKsC. Protein expression and purification of *N*-His_6_-tagged PKsC were carried out using the same procedures as those for MucE. Measurements of protein concentrations were performed with the Bradford assay using bovine serum albumin as a standard. The purified proteins were stored at − 80 °C in 20 mM Tris–HCl (pH 7.5) with 20% glycerol.

### Enzymatic assays of MucE

A holo-MucE assay was conducted at 37 °C for 1 h in 200 μL reaction mixture containing 1 μM holo-MucE, 1 μM PKsC, 100 mM Tris–HCl (pH7.5), 0.1 mM Malonyl-CoA, and 0.25 mM d-M-283-SNAC or l-M-283-SNAC. The reaction mixtures were extracted with 200 μL EtOAc twice, evaporated *in vacuum* and dissolved in 200 μL methanol for HPLC detection at a wavelength of 254 nm. HPLC detection of the MucE enzymatic assays was carried out using a C18 column (4.6 × 250 mm, 5 μm, Apollo, Alltech, Lexington, Kentucky, USA) on a Shimadzu HPLC system developed with solvent A (H_2_O with 0.1% (v/v) formic acid) and B (acetonitrile) at a flow rate of 1.0 mL/min. The percentage of acetonitrile was kept at 20% over 0–5 min, changed from 20 to 90% over 5–25 min, and from 90 to 100% over 25–30 min.

### Expression and activity testing of the MucF homologues

For in vitro characterization, the 0.5-kb *mucF*_*35*_ gene was amplified with primer pair pET-mucF-F/pET-mucF-R and inserted into pET28a at the *Nco*I/*Xho*I sites to generate pET28a-MucF_35_, which was then verified by sequencing and transformed into *E. coli* Rosetta 2(DE3)pLysS to construct *E. coli* Rosetta 2(DE3)pLysS/pET28a-MucF_35_. A single transformant was inoculated into TB broth (Thermo Fisher Scientific, USA) with 50 μg/mL kanamycin, 50 μg/mL chloramphenicol, and cultured overnight at 37 °C, 220 rpm. The overnight culture was used to inoculate in 1 L TB broth with the same antibiotics at 1:1000 dilution and incubated at 37 °C, 220 rpm until OD_600_ reached 0.6. The cells were then induced with 0.5 mM IPTG, cultured at 18 °C for a further 16 h, harvested by centrifugation, and re-suspended in 10 mL reaction buffer (50 mM Tris–HCl, 150 mM NaCl, 10% glycerol, pH 8.0) supplemented with 0.5 mg/mL lysozyme and 0.5 mM PMSF. After sonication at 4 °C, 96 μL lysate was mixed with 4 μL RTC solution (6.6 mM, 80% EtOH) and incubated at 37 °C for 30 min. The reaction was terminated by the addition of 1 μL acetic acid, extracted twice with 200 μL EtOAc, then evaporated *in vacuum* and dissolved in 200 μL methanol for LC–MS analysis. *E. coli* Rosetta 2(DE3)pLysS/pET28a was treated in the same procedure as a negative control. The encoding gene of MucF_Sgo_ was codon optimized and inserted into pET28a and its function was verified using the same method as *E. coli* Rosetta 2(DE3)pLysS/pET28a-MucF_35_.

For in vivo testing, *mucF*_*35*_ was codon optimized, synthesized, and inserted into pEXT06 (containing *mucA*–*E* for synthesizing RTCs in *E. coli*) to generate pEXT06-MucF_35_ and transformed into *E. coli* BAP1 to generate *E. coli* BAP1::*mucA*–*E*/MucF_35_. A single transformant was inoculated into LB with 1% glucose, 50 μg/mL chloramphenicol, and cultured at 37 °C, 220 rpm for 6 h. The culture was then inoculated into 50 mL LB with 1% glucose, 50 μg/mL chloramphenicol at 1:50 dilution, and incubated at 37 °C, 220 rpm until OD_600_ reached 0.4–0.6. Expression of protein was induced by the addition of IPTG at a final concentration of 0.2 mM and further cultured at 30 °C, 220 rpm for 12–14 h. After harvesting cells by centrifugation, 500 μL ddH_2_O was added to 0.1 g pellets, mixed with glass beads, and vortex 3 times for 15 min at room temperature. The lysates were then extracted twice with an equal volume of EtOAc (added with 1% acetic acid), evaporated *in vacuum*, and dissolved in 200 μL methanol for LC–MS analysis. *E. coli* BAP1::*mucA*–*E* (*E. coli* BAP1 containing unmodified pEXT06) was treated in the same procedure as a negative control. The construction of expression vectors and functional verification of other MucF homologues were also processed in the same way as the in vivo testing of *mucF*_*35*_ (except for *mucF*_*Sgo*_ whose insertion into pEXT06 failed; therefore, its function was characterized in vitro as described above).

Agilent 1290/6470 Triple-Quadrupole LC/MS system (Santa Clara, CA, USA) was used to analyze the reactions. The LC/TQ used a Jet Stream electrospray ionization source operated in the positive ionization mode. Samples were analyzed using a C18 column (2.1 × 100 mm, 2.7 μm, Agilent Technology Inc, Santa Clara, CA, USA) developed with solvent A (H_2_O) and B (acetonitrile) both with 0.1% (v/v) formic acid at a flow rate of 0.3 mL/min. The percentage of acetonitrile was changed from 10 to 100% over 0–15 min, kept at 100% over 15–20 min, and equilibrated at 5% over 20–25 min.

### Chemical syntheses and separation of d/l-M-283-SNAC

The detailed synthesis procedures of *d-M-283*, *l-M-283*, *d-M-436*, *l-M-436*, *M-307*, *M-283-SNAC*, *d-Leu-SNAC*, and *M-155* were described in Additional file 1. For the synthetic compounds, LC–MS analyses were performed on an Agilent 1260/6460 Triple-Quadrupole LC/MS system (Santa Clara, CA, USA) with an electrospray ionization source using a C18 column (4.6 × 250 mm, 5 μm, Apollo, Alltech, Lexington, Kentucky, USA). HR-ESI–MS was performed on an Agilent 1260 HPLC/6520 QTOF-MS instrument (Santa Clara, CA, USA). NMR spectra were recorded on a Bruker-500 NMR spectrometer (Billerica, MA). For the comparison between (*S*)-1-(naphthalen-2-yl)ethan-1-amine derivatives of isolated *M-283* and synthesized standards *d/l-M-436*, HPLC detection was carried out on a Shimadzu HPLC system using a C18 column (4.6 × 250 mm, 5 μm, Apollo, Alltech, Lexington, Kentucky, USA) developed with solvent A (H_2_O with 0.1% (v/v) formic acid) and B (acetonitrile) at a flow rate of 1.0 mL/min. The percentage of acetonitrile was kept at 40% over 0–3 min, changed from 40 to 70% over 3–6 min and from 70 to 76% over 6–21 min, kept at 76% over 21–25 min, changed from 76 to 90% over 25–26 min and from 90 to 100% over 26–28 min, and kept at 100% over 28–34 min.

The synthetic *M-283-SNAC* is a mixture of two enantiomers. Chiral separation of *M-283-SNAC* was performed on an Agilent 1260 series HPLC system using a Chiralpak AD-H column (4.6 × 250 mm, 5 μm, Daicel, Tokyo, Japan) developed with solvent A (isopropanol) and B (hexane) at a flow rate of 1.0 mL/min. The percentage of hexane was kept at 95%. Circular dichroism (CD) measurements of the two enantiomers in methanol were then carried out on a Chirascan spectrometer (Applied Photophysics Ltd., UK) using quartz cells with a path length of 1 mm at 25 °C. Scans were conducted between wavelengths of 190 and 400 nm with a bandwidth of 1 nm, 1-nm step resolution, 100 nm/min scan speed, and 2-s response time. Assignment of the two enantiomers were performed by comparing their CD spectra with the calculated electronic circular dichroism (ECD) spectra of *d-M-283-SNAC* and *l-M-283-SNAC* (Additional file 1, Fig. S22), which were carried out with Conflex 7.0a, Gaussian9, and Shermo 2.3 as described [49].

### Sequence and phylogenetic analyses

BLASTP search (https://blast.ncbi.nlm.nih.gov/Blast.cgi) with default parameters (nr database, BLOSUM62 matrix, expect threshold = 0.05, word size = 6) was used to assign protein functions in the *muc* gene cluster (Additional file 1, Table S1) or to identify homologous proteins. Specifically, MucF-like proteins were identified using the sequence of MucF_35_ as a query, and results with coverage above 90%, and identity above 50% were retrieved. AntiSMASH (v5.1.2) [50] (https://antismash.secondarymetabolites.org/#!/start) was used to analyze the genome sequences of strains containing the MucF-like proteins to investigate whether they have the *muc* gene cluster (Additional file 1, Table S3). TAS1-like proteins were identified using the protein sequence of TAS1 from *P. oryzae* 70–15 as a probe, and results with coverage above 80%, and identity above 30% were retrieved. The genome sequences of corresponding strains were also analyzed by antiSMASH to confirm the integrity of *TAS1* and the results were manually checked (truncated sequences or sequences containing unrelated domains were discarded; considering that the TAS1 homologous sequences in plant genomes may be contamination from fungi, three sequences from *Quercus suber* HL8 were excluded) (Additional file 1, Table S4).

For the phylogenetic analysis of MucD-C, BGCs containing different subtypes of C domains were downloaded from MIBiG (v2.0) [51] (https://mibig.secondarymetabolites.org/) and analyzed by HMMER (v3.3.2) [52] (https://www.ebi.ac.uk/Tools/hmmer/) to obtain the C-domain sequences. All of the sequences were provided as a source data file. Multiple sequence alignments were generated using MUSCLE [53] (https://www.ebi.ac.uk/Tools/msa/muscle/) with default parameters and manually trimmed. The output file was converted to.phy format by Mesquite (v3.61) and used as the input file for the phylogenetic analysis carried out using PhyML 3.0 [54] (http://www.atgc-montpellier.fr/phyml) with SMS (v1.8.4). The phylogenetic tree was visualized using iTOL (v6) [55] (https://itol.embl.de/).

To explore the distribution of *muc*-like BGC, Cblaster (v1.3.12) [56] was performed using the MucA–J protein sequences from *S. mutans* 35 as queries with the following parameters: cblaster search -qf mucAJ.fasta -mi 20 -p plot.html -o summary.csv -s session.json. The results of all the retrieved gene clusters were extracted through the extract_clusters parameter, clusters containing *mucA*–*E* homologous genes were manually checked, and the corresponding genomes were further analyzed by antiSMASH. Multiple nucleotide sequence alignments for the 63 resultant BGCs (ranging from *mucA* to *mucE*) were generated using MAFFT [53] (https://www.ebi.ac.uk/Tools/msa/mafft/) with default settings. Phylogenetic analysis of the resulting output file was carried out similarly to MucD-C.

## Supplementary Information

**Additional file 1.** Supplementary Tables, Supplementary Figures.

## Data Availability

Data supporting the findings of this work are available within the paper and the Additional file 1. The genome sequence of *S. mutans* B30 has been deposited into GenBank with the accession number JAGEVH000000000.
