# Supplementary material for: Human-associated bacteria adopt an unusual route for synthesizing 3-acetylated tetramates for environmental adaptation
Source: Microbiome. 2023 May 5;11:97. doi: 10.1186/s40168-023-01548-4 (PMC10161427; doi:10.1186/s40168-023-01548-4)
Supplement: Supplementary file 2 — Additional file 1. Supplementary Tables, Supplementary Figures. [file 40168_2023_1548_MOESM1_ESM.zip › SI.pdf]

Supporting Information for

Human-associated bacteria adopt an unusual route for synthesizing 3-acetylated tetramates for environmental adaptation

Yuwei Zhang,[a,b,#] Ge Liao,[c,#] Min Wang,[a,d,#] Zhao Zhang,[a,e,#] Liwei Liu[e], Yuqin Song[a], Dacheng Wang[a], Tingting Hao,[a,b] Jie Feng,[a,b] Bin Xia[f], Yixiang Wang[g], Xiaoyu Tang[c*], Yihua Chen[a,b*]

a State Key Laboratory of Microbial Resources, Institute of Microbiology, Chinese Academy of Sciences, Beijing 100101, China

b University of Chinese Academy of Sciences, Beijing 100049, China

c Institute of Chemical Biology, Shenzhen Bay Laboratory, Shenzhen 518132, China

d School of Biotechnology and Health Sciences, Wuyi University, Jiangmen 529020, Guangdong, China

e Renal Division, Peking University First Hospital, Peking University Institute of Nephrology, Beijing 100034, China

f Department of Marine Pharmacy, College of Food and Pharmaceutical Sciences, Ningbo University, Ningbo 315211, China

g Department of Pediatric Dentistry, Peking University School and Hospital of Stomatology, Beijing 100081, China

h Central Laboratory Peking University School and Hospital of Stomatology, Beijing 100081, China

[#]These authors contributed equally.

*Corresponding authors

*Yihua Chen

Email: chenyihua@im.ac.cn

*Xiaoyu Tang

Email: xtang@szbl.ac.cn

This PDF file includes:

- Tables S1, S2, and S4 to S6
- Figures S1 to S22
- Legend for Table S3
- SI References

Other supporting materials for this manuscript include the following: Tables S3

Supplementary Tables

Table S1. Proteins encoded by the *muc* BGC and their homologues

Protein	Homo logues	Predicated function	Organism	Coverage /Identity	Accession number
MucA	RtcA	3-oxoacyl-ACP synthase	*Lactobacillus reuteri*	98%; 57.34%	KEK15823.1
	PhlA	hydroxymethylglutaryl-CoA synthase	*Pseudomonas fluorescens*	98%; 30.75%	BAD00178.1
MucB	RtcC	thiolase family protein	*L. reuteri*	98%; 65.58%	WP_035152893.1
	PhlC	thiolase family protein	*P. fluorescens*	98%; 34.84%	BAD00179.1
MucC	RtcB	OB-fold domain-containing protein	*L. reuteri*	97%; 68.97%	WP_035152895.1
	PhlB	OB-fold domain-containing protein	*P. fluorescens*	94%; 37.59%	AAM27407.1
MucD	RtcN	NRPS	*L. reuteri*	99%; 47.82%	AJO68335.1
MucE	RtcK	PKS	*L. reuteri*	99%; 52.8%	AJO68346.1
MucF		HXXEE domain-containing protein	*Streptococcus macedonicus*	99%; 57.49%	WP_099390897.1
MucG		TetR/AcrR family transcriptional regulator	*Streptococcus troglodytae*	91%; 98.89%	WP_128833890.1
MucH		TetR/AcrR family transcriptional regulator	*Streptococcus macacae*	99%; 98.33%	WP_003081357.1
MucI		DHA2 family efflux MFS transporter permease subunit	*S. troglodytae*	99%; 99.83%	WP_128833889.1
MucJ		small multi-drug export protein	*S. macacae*	99%; 97.32%	WP_003082389.1

Table S2. Distribution of strains harboring *muc*-like BGCs.

Strain	Host; isolation source	Location	Collection date
Streptococcus mutans			
24	*Homo sapiens*; oral cavity	Iceland	1990
B04Sm5	*Homo sapiens*; carious lesion	USA	2014
KK23	*Homo sapiens*; enamel caries of a child	Germany	2013
B05Sm11	*Homo sapiens*; oral cavity	USA	2003
M2A	*Homo sapiens*; oral cavity	UK	2006
B115SM-A	*Homo sapiens*; oral cavity	USA	2009
AC4446	*Homo sapiens*; infective endocarditis	Germany	2013
35	*Homo sapiens*; oral cavity	China	2015
B30	*Homo sapiens*; oral cavity	China	2015
2VS1	*Homo sapiens*; oral cavity	Brazil	2013
NLML4	*Homo sapiens*; oral cavity	UK	2001
B	*Homo sapiens*; oral cavity	Iceland	1990
G123	*Homo sapiens*; oral cavity	UK	2006
14D	*Homo sapiens*; oral cavity	Iceland	1990
HM	*Homo sapiens*; infective endocarditis	Japan	2017
W6	*Homo sapiens*; oral cavity	UK	2006
NN2025	*Homo sapiens*; oral cavity; Dental caries	Japen	2002
B06Sm2	*Homo sapiens*; oral cavity	USA	2003
NLML9	*Homo sapiens*; oral cavity	UK	2001
SM6	*Homo sapiens*; oral cavity	China	2001
NCTC10832	*Homo sapiens*; dental abscess	UK	1900/1972
M21	*Homo sapiens*; oral cavity	UK	2006
B082SM-A	*Homo sapiens*; oral cavity	USA	2008
B107SM-B	*Homo sapiens*; oral cavity	USA	2009
1002_SMUT	*Homo sapiens*; from ICU	USA	2014
1006_SMUT	*Homo sapiens*; from ICU	USA	2014
B07Sm2	*Homo sapiens*; oral cavity	USA	2003
P1	*Homo sapiens*; root canal	Brazil	2015
P6	*Homo sapiens*; root canal	Brazil	2015
S1	*Homo sapiens*; root canal	Brazil	2015
S4	*Homo sapiens*; root canal	Brazil	2018
UAB-12	*Homo sapiens*; dental caries plaque	USA	2007
UAB-20	*Homo sapiens*; dental caries plaque	USA	2009
UAB-24	*Homo sapiens*; dental caries plaque	USA	2007
UAB-35	*Homo sapiens*; dental caries plaque	USA	2010
UAB-40	*Homo sapiens*; dental caries plaque	USA	2011
BgEED33	*Homo sapiens*; duodenal aspirate	USA	2019
**Other *Streptococcus***			
S. troglodytae TKU31	Chimpanzee; oral cavity	Japan	2009/2010
S. macacae NCTC11558	Monkey (Macaque); Dental Plaque	UK	1900/1983

S. sp. HMSC068F04	*Homo sapiens*; sputum	USA	2016
S. orisasini SH06	*Equus caballus*; feces of healthy thoroughbred	Japan	2015
S. *lutetiensis* BR_UFV01	Ruminal fluid from bovine	Brazil	2015
Lactiplantibacillus			
L. plantarum 4_3	fermented soybean	China	2011
L. plantarum LQ80	pig feed from feed plant	Japan	2002
L. plantarum Tw226	*Odontesthes bonariensis* (Pejerrey)	Argentina	2010
L. paraplantarum OSY-TC318	Turkish cheese	USA	2016
L. plantarum BGM37	Brine from olive fermentation	USA	2011
Limosilactobacillus			
L. reuteri LTH2584	Type II sourdough	Germany	1988
L. reuteri TMW1.656	Type II sourdough	Germany	1998
L. reuteri TMW1.112	Type II sourdough	Germany	1998
L. reuteri LTH5448	Type II sourdough	Germany	1998
L. reuteri LTM_Jesus_5_1	Lion-tailed Macaque	USA	2014
Lactococcus			
L. hircilactis DSM 28960	goat milk	not collected	not collected
Bifidobacterium			
B. kashiwanohense PV20-2	*Homo sapiens*; feces	Kenya	2010
B. longum subsp. *infantis* UBBI-01	fermented food	India	2014
Bifidobacteriaceae *bacterium* NR016	*Homo sapiens*; vaginal fluid	Kenya	2012
Bacillus			
B. wiedmannii AFS078926	soybean	USA	2014
B. cereus AFS079194	plant core	USA	2014
B. cereus AFS046754	plant core	USA	2014
B. cereus IZSPB_BC561A	ice cream	Italy	2019
B. thuringiensis 871Blist1	surface of a grape leaf from a vineyard	Russia	2019
B. pseudomycoides AFS069829	corn	USA	2014
Priestia			
P. megaterium 22-2	not applicable	China	2016

Table S4. Distribution of strains harboring *TAS1*-like genes.

Accession	Strain	Host; isolation source	Location	Collection date
XP_003713020.1	*Pyricularia oryzae* 70-15	pathogenic on rice		
KAH8848388.1	*Pyricularia oryzae* K23/123	Eleucine coracana	Kenya	2000
KAH9429665.1	*Pyricularia oryzae* E34	Eleusine coracana	Japan	1976
ELQ64701.1	*Pyricularia oryzae* P131	*Oryza sativa* L. ssp. *japonica*; infected leaves	Japan	
ELQ37655.1	*Pyricularia oryzae* Y34	*Oryza sativa* L. ssp. *indica*; infected leaves	China	
XP_030987666.1	*Pyricularia grisea* NI907	*Digitaria sanguinalis*	Japan	1974
KAI1120014.1	*Nemania abortiva* FL1152	not applicable	USA	missing
KAI1318605.1	*Xylariaceae* sp. FL0255	not applicable	USA	missing
XP_031934908.1	*Aspergillus pseudonomiae* CBS 119388	not applicable	missing	missing
XP_015404840.1	*Aspergillus nomiae* NRRL 13137	wheat	USA	1987
KAB8076255.1	*Aspergillus leporis* CBS 151.66	Dung of *Lepus townsendii*	USA	missing
KAE8378832.1	*Aspergillus bertholletiae* IBT 29228	not applicable	missing	missing
XP_022387672.1	*Aspergillus bombycis* NRRL26010	silkworm rearing house	Japan	1987
XP_031925180.1	*Aspergillus caelatus* CBS 763.97	not applicable	missing	missing
XP_028507553.1	*Alternaria arborescens* FERA 675	apple; infected leaves	missing	missing
OWY51330.1	*Alternaria alternata* Z7	*Citrus suavissima*	China	2011
RYN38034.1	*Alternaria arborescens* RGR 97.0013	apple; infected leaves	missing	missing
RYN96158.1	*Alternaria tenuissima* FERA 648	apple; infected leaves	missing	missing
PIG83113.1	*Aspergillus arachidicola* CBS 117610	Arachis glabrata	Argentina	2004
XP_018383119.1	*Alternaria alternata* SRC1lrK2f	passive coal mine drainage treatment systems	missing	missing
KAH6846806.1	*Alternaria alternata* MPI-PUGE-AT-0064	*Arabidopsis thaliana*	missing	missing
RYN72957.1	*Alternaria alternata* FERA 1177	apple; infected leaves	missing	missing
RYN38103.1	*Alternaria tenuissima* FERA 1082	apple; infected leaves	missing	missing
RYN63383.1	*Alternaria tenuissima* FERA 24350	apple; infected leaves	missing	missing
XP_038789627.1	*Alternaria burnsii* CBS107.38	*Cuminum cyminum*	India	1938
KAB2105161.1	*Alternaria gaisen* FERA 650	apple; infected leaves	missing	missing
RYO52509.1	*Alternaria tenuissima* FERA 1166	apple; infected leaves	missing	missing
RYN22078.1	*Alternaria tenuissima* FERA 1164	apple; infected leaves	missing	missing
XP_003660956.1	*Thermothelomyces thermophilus* ATCC 42464	not applicable		
RAR03424.1	*Stemphylium lycopersici* CIDEFI 213	*Solanum lycopersicum* cv. Elpida	Argentina	2011
KAE8157711.1	*Aspergillus tamarii* CBS 117626	not applicable	missing	missing
RAR05690.1	*Stemphylium lycopersici* CIDEFI 212	*Solanum lycopersicum* cv. Elpida	Argentina	2010

XP_031919586.1	*Aspergillus pseudotamarii* CBS 117625	not applicable	missing	missing
CEJ93579.1	*Torrubiella hemipterigena*	not applicable		
XP_018700739.1	*Cordyceps fumosorosea* ARSEF 2679	*Popillia japonica*	Portugal	1988
XP_006667652.1	*Cordyceps militaris* CM01	*Cordyceps militaris*		
ATY60283.1	*Cordyceps militaris* ATCC 34164	butterfly pupa	missing	missing
XP_044717203.1	*Hirsutella rhossiliensis* HR02	missing	missing	missing
XP_007868147.1	*Gloeophyllum trabeum* ATCC 11539	not applicable		
KIK00478.1	*Laccaria amethystina* LaAM-08-1	not applicable		
KDR67250.1	*Galerina marginata* CBS 339.88	missing	missing	not collected
KIK03064.1	*Laccaria amethystina* LaAM-08-1	not applicable		
KAF8517297.1	*Hysterangium stoloniferum* HS.BST	not applicable	missing	missing
KAH7015653.1	*Ilyonectria destructans* MPI-CAGE-AT-0134	*Arabidopsis thaliana*	missing	missing
KAH6983355.1	*Ilyonectria* sp. MPI-CAGE-AT-0026	not applicable	missing	missing
PPQ97880.1	*Gymnopilus dilepis* SRW20	oak tree sawdust	USA	2013
XP_007815200.1	*Metarhizium acridum* CQMa 102	not applicable		
KAG8415777.1	*Metarhizium acridum* ARSEF 324	*Austracris guttulosa*	Australia	1979
KAH7241374.1	*Fusarium tricinctum* MPI-SDFR-AT-0068	*Arabidopsis thaliana*	France	missing
KAG8418963.1	*Metarhizium acridum* ARSEF 324	*Austracris guttulosa*	Australia	1979
XP_044725459.1	*Hirsutella rhossiliensis* HR02	missing	missing	missing
KAH0593838.1	*Metarhizium humberi* ESALQ1638	native vegetation	Brazil	2012
KJK94653.1	*Metarhizium anisopliae* BRIP 53284	not applicable		
KID84426.1	*Metarhizium guizhouense* ARSEF 977	*Melolontha melolontha*	France	1983
KID66114.1	*Metarhizium anisopliae* ARSEF 549	unknown	Brazil	1980
XP_045959157.1	*Truncatella angustata* MPI-SDFR-AT-0073	*Arabidopsis thaliana*	France	missing
KJK81393.1	*Metarhizium anisopliae* BRIP 53293	soil	Australia	
KIL88590.1	*Fusarium avenaceum* Fa05001	*Hordeum vulgare* L.	Finland	2005
KAH6950151.1	*Fusarium avenaceum* MPI-SDFR-AT-0044	*Arabidopsis thaliana*	missing	missing
ODA79169.1	*Drechmeria coniospora* ATCC 96282	nematode; whole body	Sweden	missing
XP_035366400.1	*Lasiodiplodia theobromae* AM2As	*Theobroma cacao*; young plant	Indonesia	2014
KAF9636613.1	*Lasiodiplodia theobromae* CSS-01s	*Vitis vinifera*	China	2009
TQB70024.1	*Monascus purpureus* HQ1	Wine factory	China: Hubei	2010
KAI0151338.1	*Pestalotiopsis* sp. NC0098	*Acer rubrum* (Sapindaceae)	USA	2012
KJZ80421.1	*Hirsutella minnesotensis* 3608	soybean cyst nematode; soil	China	2006
KAF4991222.1	*Fusarium decemcellulare* NRRL 13412	*Coffea* sp.	Dominican Republic	missing

KAF4631120.1	*Cudoniella acicularis* DSM 108380	deciduous deadwood	Germany	2017
EWZ97080.1	*Fusarium oxysporum* f. sp. lycopersici MN25	not applicable		
KAF7554762.1	*Cylindrodendrum hubeiense* IHI 201604	deciduous deadwood	Germany	2016
KAI0185232.1	*Xylaria flabelliformis* NC1011	not applicable	USA	missing
KAF5139466.1	*Metarhizium anisopliae* JEF-290	Insect; whole organism	South Korea	2015
XP_040656630.1	*Drechmeria coniospora* ARSEF 6962	missing	Sweden	1987
KAI0544201.1	*Xylaria curta* CBS 114988	not applicable	missing	missing
KAH7159175.1	*Fusarium* sp. MPI-SDFR-AT-0072	*Arabidopsis thaliana*	missing	missing
KAI0397031.1	*Xylariaceae* sp. FL0594	not applicable	USA	missing
KAG7407204.1	*Fusarium oxysporum* f. sp. rapae	*Brassica rapa*	Japan	2010
KAI0859890.1	*Xylaria cubensis* CBS 116.85	not applicable	missing	missing
XP_046105152.1	*Ilyonectria robusta* PMI_751	not applicable	missing	missing
KAI0902873.1	*Kretzschmaria deusta* CBS 826.72	not applicable	missing	missing
KAF4471968.1	*Fusarium albosuccineum* NRRL 20459	tree	missing	missing
KAI1734901.1	*Xylaria scruposa* CBS 123580	not applicable	missing	missing
KAF2733364.1	*Polyplosphaeria fusca* CBS 125425	missing	missing	missing
XP_040653756.1	*Drechmeria coniospora* ARSEF 6962	missing	Sweden	1987
KDQ18939.1	*Botryobasidium botryosum* FD-172 SS1	not applicable		
ODA77312.1	*Drechmeria coniospora* ATCC 96282	nematode	Sweden	missing
KAF9528634.1	*Crepidotus variabilis* CBS 506.95	not applicable	missing	missing
APX43999.1	*Pestalotiopsis microspora* NK17	*Taxus wallachiana*; endophytic fungi	China	
KZP16709.1	*Fibularhizoctonia* sp. CBS 109695	termite symbiont		
KZP12421.1	*Fibularhizoctonia* sp. CBS 109695	termite symbiont		
KZP16701.1	*Fibularhizoctonia* sp. CBS 109695	termite symbiont		
KAB2573376.1	*Lasiodiplodia theobromae* LA-SOL3	*Vitis vinifera*; cultivar Red Globe	Peru	2012/2013
KAF9071084.1	*Rhodocollybia butyracea* AH 40177	Humus (*Castanea sativa*)	missing	missing
TQV99846.1	*Cordyceps javanica* IJ1G	*Spodoptera litura*; oriental leaf worm moth	missing	missing
OAA78214.1	*Akanthomyces lecanii* RCEF 1005	Lepidopteran; larva	China	2006
KAI0190564.1	*Astrocystis sublimbata* CBS 130006	not applicable	missing	missing
OAA48197.1	*Beauveria brongniartii* RCEF 3172	*Locusta migratoria manilensis*	China	2006
XP_033383750.1	*Aaosphaeria arxii* CBS 175.79	not applicable	missing	missing

Table S5. Strains and plasmids used in this study.

Strains/Plasmids	Characteristics	Sources
**Strains**		
S. mutans 35	Wild type	1
S. mutans UA140	Wild type	2
S. mutans UM	Heterologous expression of mutanocyclin in *S. mutans* UA140	This study
S. mutans UM UA159	Heterologous expression of mutanocyclin in *S. mutans* UA159	1
S. mutans UM *ΔmucD*	*mucD* in-frame deletion *mutant*	This study
S. mutans UM *ΔmucE*	*mucE* in-frame deletion *mutant*	This study
S. mutans UM *ΔmucF-J*	*mucF-J* in-frame deletion *mutant*	This study
S. mutans UM *mucD**(H135V)	*mucD* C domain *mut*agenesis	This study
S. mutans UM *mucD**(D139N)	*mucD* C domain *mut*agenesis	This study
Bacillus subtilis sp. 168	Wild type	3
E. coli JM109	Host strain for recombinant cloning	Promega
E. coli BAP1	Contains *sfp* and was used for protein/pathway expression	4
E. coli BL21(DE3)	Host strain for protein expression	Novagen
E. coli BAP1/pET28a-MucE	MucE expression strain	This study
E. coli BL21(DE3)/pET28a-PKsC	PKsC expression strain	This study
E. coli Rosetta 2(DE3)pLysS	Host strain for protein expression	Novagen
E. coli Rosetta 2(DE3)pLysS/pET28a	*E. coli* Rosetta 2(DE3)pLysS containing pET28a	This study
E. coli Rosetta 2(DE3)pLysS /pET28a-MucF$_{35}$	MucF$_{35}$ expression strain	This study
E. coli Rosetta 2(DE3)pLysS /pET28a-MucF$_{Sgo}$	MucF$_{Sgo}$ expression strain	This study
E. coli BAP1::*mucA-E*	*mucA-E* expression strain used for RTC producing	This study
E. coli BAP1::*mucA-E*/MucF$_{35}$	RTC producing/MucF$_{35}$ expression strain	This study
E. coli BAP1::*mucA-E*/MucF$_{Ssp}$	RTC producing/MucF$_{Ssp}$ expression strain	This study
E. coli BAP1::*mucA-E*/MucF$_{Smac}$	RTC producing/MucF$_{Smac}$ expression strain	This study
E. coli BAP1::*mucA-E*/MucF$_{SHM}$	RTC producing/MucF$_{SHM}$ expression strain	This study
E. coli BAP1::*mucA-E*/MucF$_{Slu}$	RTC producing/MucF$_{Slu}$ expression strain	This study
E. coli BAP1::*mucA-E*/MucF$_{Sors}$	RTC producing/MucF$_{Sors}$ expression strain	This study
E. coli BAP1::*mucA-E*/MucF$_{Ana}$	RTC producing/MucF$_{Ana}$ expression strain	This study
E. coli BAP1::*mucA-E*/MucF$_{Aor}$	RTC producing/MucF$_{Aor}$ expression strain	This study
E. coli BAP1::*mucA-E*/MucF$_{Smas}$	RTC producing/MucF$_{Smas}$ expression strain	This study
E. coli BAP1::*mucA-E*/MucF$_{Swi}$	RTC producing/MucF$_{Swi}$ expression strain	This study
E. coli BAP1::*mucA-E*/MucF$_{Sorr}$	RTC producing/MucF$_{Sorr}$ expression strain	This study
E. coli BAP1::*mucA-E*/MucF$_{Str}$	RTC producing/MucF$_{Str}$ expression strain	This study
E. coli BAP1::*mucA-E*/MucF$_{140}$	RTC producing/MucF$_{140}$ expression strain	This study
E. coli BAP1::*mucA-E*/MucF$_{159}$	RTC producing/MucF$_{159}$ expression strain	This study
E. coli BAP1::*mucA-E*/MucF$_{Sorl}$	RTC producing/MucF$_{Sorl}$ expression strain	This study
E. coli BAP1::*mucA-E*/MucF$_{Sla}$	RTC producing/MucF$_{Sla}$ expression strain	This study
E. coli BAP1::*mucA-E*/MucF$_{Spa}$	RTC producing/MucF$_{Spa}$ expression strain	This study
**Plasmids**		
pET28a	Kan^r, T7 promoter	Novagen
pIFDC2	Amp^r, IFDC2 cassette	5
pZX9	*xylS1$_P$* promoter containing vector	6

pET28a-MucE	Kan^{r}, MucE expression vector	This study
pET28a-PKsC	Kan^{r}, PKsC expression vector	This study
pEXT06	Chl^{r}, containing *mucA-E*	7
pET28a-MucF$_{35}$	Kan^{r}, MucF$_{35}$ expression vector	This study
pET28a-MucF$_{Sgo}$	Kan^{r}, MucF$_{Sgo}$ expression vector	This study
pEXT06-MucF$_{35}$	Chl^{r}, *mucA-E* and MucF$_{35}$ expression vector	This study
pEXT06-MucF$_{Ssp}$	Chl^{r}, *mucA-E* and MucF$_{Ssp}$ expression vector	This study
pEXT06-MucF$_{Smac}$	Chl^{r}, *mucA-E* and MucF$_{Smac}$ expression vector	This study
pEXT06-MucF$_{SHM}$	Chl^{r}, *mucA-E* and MucF$_{SHM}$ expression vector	This study
pEXT06-MucF$_{Slu}$	Chl^{r}, *mucA-E* and MucF$_{Slu}$ expression vector	This study
pEXT06-MucF$_{Sors}$	Chl^{r}, *mucA-E* and MucF$_{Sors}$ expression vector	This study
pEXT06-MucF$_{Ana}$	Chl^{r}, *mucA-E* and MucF$_{Ana}$ expression vector	This study
pEXT06-MucF$_{Aor}$	Chl^{r}, *mucA-E* and MucF$_{Aor}$ expression vector	This study
pEXT06-MucF$_{Smas}$	Chl^{r}, *mucA-E* and MucF$_{Smas}$ expression vector	This study
pEXT06-MucF$_{Swi}$	Chl^{r}, *mucA-E* and MucF$_{Swi}$ expression vector	This study
pEXT06-MucF$_{Sorr}$	Chl^{r}, *mucA-E* and MucF$_{Sorr}$ expression vector	This study
pEXT06-MucF$_{Str}$	Chl^{r}, *mucA-E* and MucF$_{Str}$ expression vector	This study
pEXT06-MucF$_{140}$	Chl^{r}, *mucA-E* and MucF$_{140}$ expression vector	This study
pEXT06-MucF$_{159}$	Chl^{r}, *mucA-E* and MucF$_{159}$ expression vector	This study
pEXT06-MucF$_{Sorl}$	Chl^{r}, *mucA-E* and MucF$_{Sorl}$ expression vector	This study
pEXT06-MucF$_{Sla}$	Chl^{r}, *mucA-E* and MucF$_{Sla}$ expression vector	This study
pEXT06-MucF$_{Spa}$	Chl^{r}, *mucA-E* and MucF$_{Spa}$ expression vector	This study

Table S6. Primers used in this study.

Primers	Sequence (5′ to 3′)	Description
UA140-upF	AACTGCGCCAATCGAAAGAC	*muc* gene cluster cloning
UA140-upR-erm	GGTATACTACTGACAGCTTCTAGATTTCCTTTGGCTTTCC	
UA140-dnF-ldh	TGAGTGTTATTGTTGCTCGGCGACTTTCAGTAACAGCACT	
UA140-dnR	AACCCAAGTGTTCTTTCATAC	
UA140-upF1	CAAACTCCGCAAAACTTAAA	*S. mutans* UM* verification
orfJ-R	CCTCTTTCTCCTGCTACCTT	
bacA-F	CGTCAACAACAAGGGGAACT	
bacA-R	ACCAGGATTTGAAGGAGAAC	
orfE-F	TGATATTATTTCCTCTAAGTTGC	
orfE-R	TTTTAGGAGGATACTCATTTGG	
orfA-F	AAATGTAAAACCGCTTCTTG	
UA140-dnR1	CCAGCATAATGGTGGTAAGA	
XylRR-BsaI	GTGGTCTCACTAACTTATAGGGGTAACACTTAAA	Activation of *muc* gene cluster
XylOR-BsaI	CAGGTCTCACTCCTTTGATTTAAGTGAACAAGT	
orfA-upF	TCTTCTTGGACAATACGAAG	
orfA-upR2-BsaI	CAGGTCTCTTCCACTATAACAAATGTTAGTAATCTGTC	
orfA-dnF2-BsaI	GTGGTCTCAGGAGGGGACATGGGCAATAA	
orfA-dnR	ATCCTCTGTTCTTCCTTGCT	
orfA-upR3-BsaI	CAGGTCTCTTAGGCTATAACAAATGTTAGTAATCTGTC	
XylRR1-BsaI	GTGGTCTCACCTAACTTATAGGGGTAACACTTAAA	
DUF	GAAAGGAGAAGCCATAGAAT	*mucD* disrupted mutant
DUR	GTGGTCTCACCTAGCATTATCAGGCTGTAGTT	
DDF	CAGGTCTCTTCCAGAAGTTGGAGGGACATCAGTT	
DDF2	CAGGTCTCTTAGGAAGTTGGAGGGACATCAGTT	
DDR	ACTACTCCGCATCCATCTCC	
DSupF	CTAGAATTGTATGATCAAGG	
DSdnR	CCAGAAATCACTGCATAAAT	
EUF	CCAGGATTTGAAGGAGAACT	*mucE* disrupted mutant
EUR	TGAGTGTTATTGTTGCTCGGTTGAGTCGGAGTATTAGCATC	
EUR2	GTTCTGCCAATCTTTAGCATCTTGAGTCGGAGTATTAGCATC	
EDF	GGTATACTACTGACAGCTTCGATGCTAAAGATTGGCAGAAC	
EDF2	GATGCTAATACTCCGACTCAAGATGCTAAAGATTGGCAGAAC	
EDR	GTATTGCCTATGCCTATCAC	
ESupF	GGGAAATCCATTGATAATAC	
ESdnR	TGGCCCTGCCTTTTCAATAG	
ldhF-BsaI	CAGGTCTCTTAGGAGCAACAATAACACTCATAGC	IFDC2 cassette PCR
ermR-BsaI	GTGGTCTCATGGAAGCTGTCAGTAGTATACCTAA	
ldhF	CCGAGCAACAATAACACTCA	
ermR	GAAGCTGTCAGTAGTATACC	
CdHIUF	CTCGGTTAGAAGGAAGTA	C domain H135V mutation
CdHIUR	GTGGTCTCACCTACTCACATTAATAAAAAGAAAATA	
CdHIDF	CAGGTCTCTTCCATTGATTTGTGATATTTATAGTGC	
CdHIDR	ATCGTCCTCAGTTTGTTT	
CdHUR	GTGGTCTCAAACACTCACATTAATAAAAAGAAAATA	
CdHDF	CAGGTCTCTTGTTTTGATTTGTGATATTTATAGTGC	
CdDIUF	CTCGGTTAGAAGGAAGTA	C domain D139N mutation
CdDIUR	GTGGTCTCACCTACAAATCAAATGACTCACA	

CdDIDF	CAGGTCTCTTCCAATTTATAGTGCCTATGAAA	
CdDUR	GTGGTCTCAATTACAAATCAAATGACTCACA	
CdDDF	CAGGTCTCTTAATATTTATAGTGCCTATGAAA	
CdDDR	TCGTCCTCAGTTTGTTTG	
CdmF	CGGTAACCTTGAACTAAAGT	Point mutation sequencing
CdmR	ATTTGTCTGCCCTTATTGCT	
ldhF-MucJ	TTCCGATCATCTTTTTCGAGCAACAATAACACTCATAGC	*mucF-J* disrupted mutant
ermR1-MucF	TGTAGAGAGGGCATGATTTTGAAGMUCTCAGTAGTATACCTAA	
JUF	GGTCATAGATGGTATCCTTT	
JUR	GAAAAAGATGATCGGAACAG	
JUR2	TGTAGAGAGGGCATGATTTTGAAAAAGATGATCGGAACAG	
FDF	AAAATCATGCCCTCTCTACA	
FDF2	TTCCGATCATCTTTTTCAAAATCATGCCCTCTCTACA	
FDR	AGCCTTMUCGAGATTCTTAT	
MucJ-upF	GAGGACTTAGGCTTTTTACC	
MucF-dnR	ATGCTATTTCTTTCCCTCAA	
PKsCF	GATCCCATGGGAATCACATATGTTTTTCCAGGTCAGG	PKsC PCR
PKsCR	GATCCTCGAGGCGGGGCATTGCTTCCGCATCTTTC	
T7F	GGGAATTGTGAGCGGATAAC	MucE expression
mucER1	TGCTAGTTATTGCTCAGCGG	
mucEF2	CGCAAGCCTATCATTTCCAA	
mucER2	CGATCCTTTTTGCCCTTTAT	
mucEF3	CAACTCCAAGGGAATTAGCA	
T7R	CCTACAATTCCTGATGCTGT	
pET-mucF-F	GAAGGAGATATACCATGGATCTATTATCTAATTATTTCATG	*S. mutans* 35 MucF cloning
pET-mucF-R	GTGGTGGTGGTGCTCGAGGCCTTTAAATAAGTTTAACAG	

Supplementary Figures

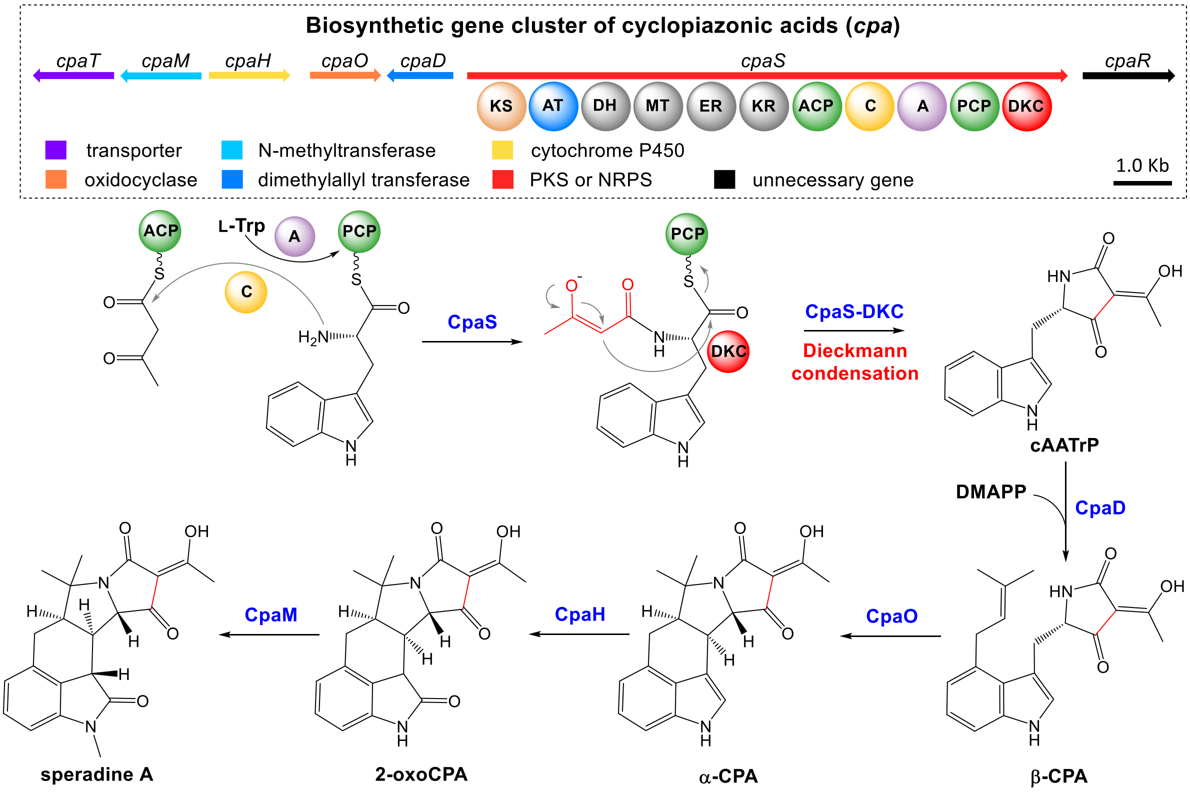

Fig. S1. Cyclopiazonic acids are 3-acetyled TACs assembled by a PKS/NRPS machinery via Dieckmann cyclization. Cyclopiazonic acid (CPA), a mycotoxin produced by several *Aspergillus* and *Penicillium* species, forms the pyrrolidine-2,4-dione ring through Dieckmann cyclase (DKC) catalyzed Dieckmann reaction[8]. In brief, CpaS-DKC domain releases the PCP-tethered linear product of PKS and NRPS (N-acetoacetyl-Trp) and catalyzes Dieckmann cyclization to generate cyclo-acetoacetyl-L-Trp (cAATrp). Afterwards, cAATrp is prenylated by CpaD with dimethylallyl pyrophosphate (DMAPP) to yield β-CPA, which is then converted to α-CPA through an oxidative cyclization mechanism catalyzed by CpaO. The conversion of α-CPA to less toxic 2-oxoCPA is catalyzed by the P450 enzyme CpaH in some strains as self-detoxification[9]. Further methylation to speradine A is catalyzed by CpaM[10].

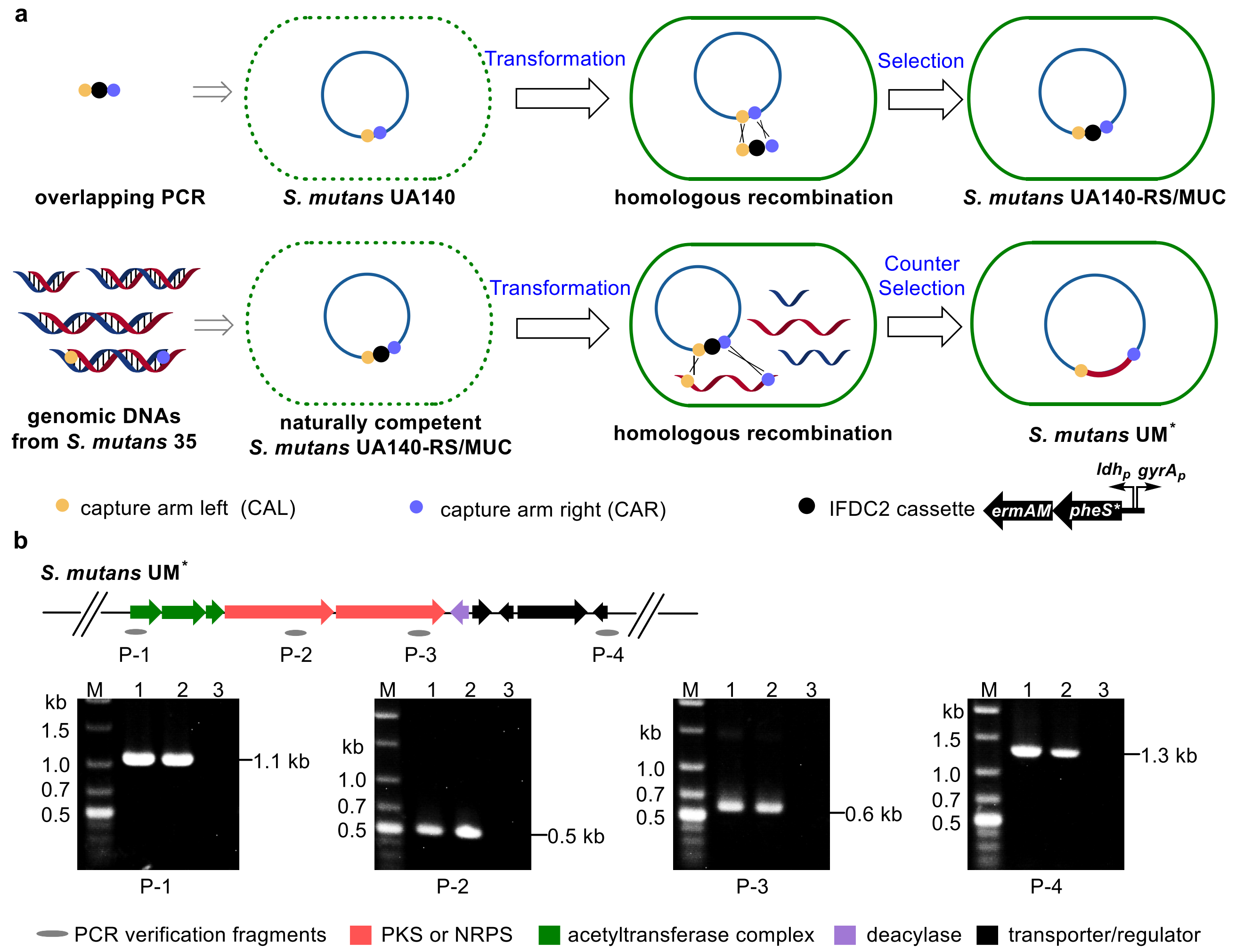

Fig. S2. Cloning the *muc* gene cluster into *S. mutans* UA140. (**a**) The *muc* gene cluster was cloned into *S. mutans* UA140 using the NabLC technique. Sequences flanking the *muc* gene cluster together with the IFDC2 cassette were ligated by overlapping PCR and transformed to *S. mutans* UA140 to construct the recipient strain *S. mutans* UA140-RS/MUC. Then the genomic DNAs of *S. mutans* 35 were transformed to *S. mutans* UA140-RS/MUC via natural competence. The *muc* gene cluster was inserted into the genome of the recipient strain by homologous recombination and the recombinant strain *S. mutans* UM* was screened using the counterselection marker. (**b**) Four different parts (marked as P-1 to P-4) of the *muc* gene cluster were selected for PCR verifications. Lane M, DNA marker; Lane 1, *S. mutans* UM*; Lane 2, positive controls using the genomic DNA of the donor strain *S. mutans* 35 as a template; Lane 3, negative controls using the genomic DNA of the recipient strain *S. mutans* UA140-RS/MUC as a template.

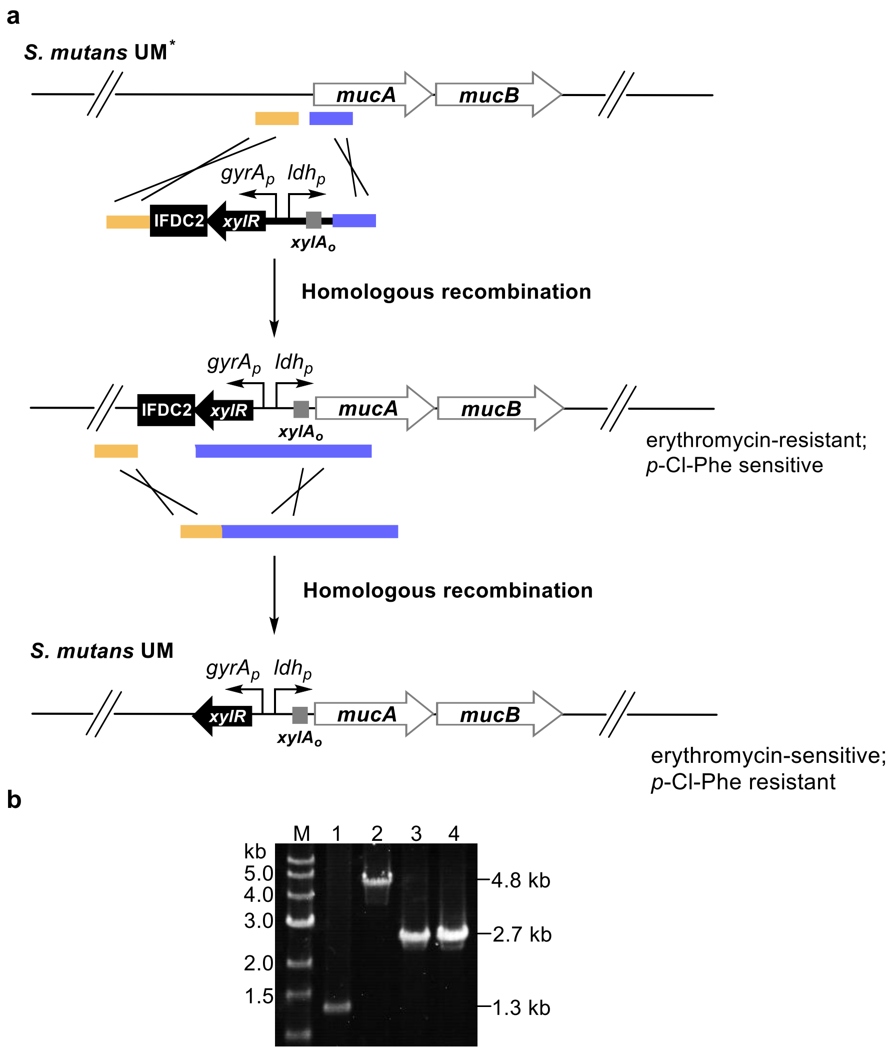

Fig. S3. Activation of the *muc* gene cluster in *S. mutans* UA140. (**a**) The *muc* gene cluster was activated in *S. mutans* UM* by inserting the xylose-inducible promoter $xylS1_P$ upstream of the gene cluster via homologous recombination to generate *S. mutans* UM. (**b**) PCR verification of the insertion of $xylS1_P$. Lane M, DNA marker; Lane 1, negative control using the genomic DNA of *S. mutans* UM* as a template; Lane 2, PCR verification of strain *S. mutans* UM*/IFDC2-$xylS1_P$; Lane 3-4, PCR verifications of *S. mutans* UM strains.

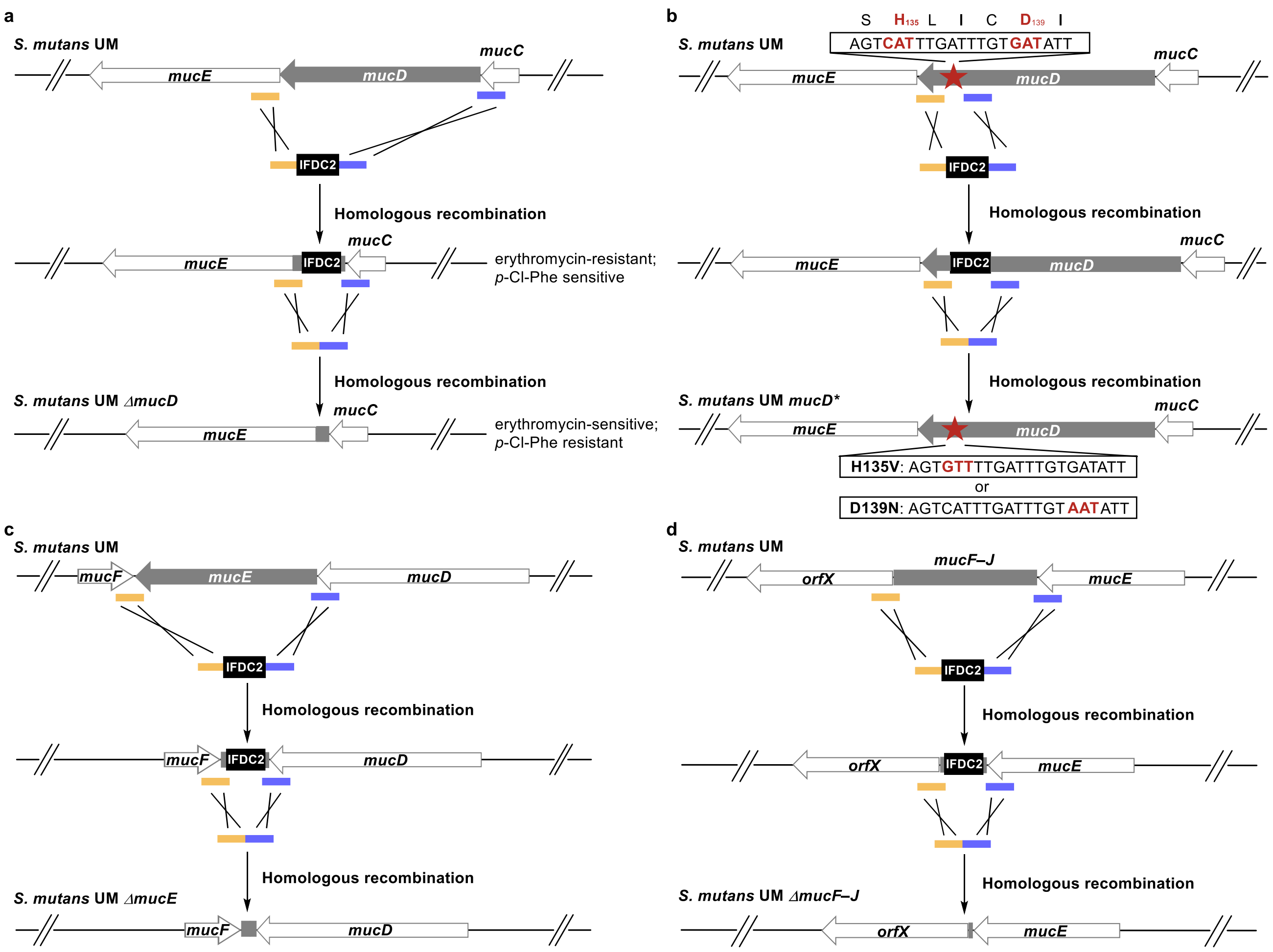

Fig. S4. Construction of *S. mutans* UM mutants. (**a**) Construction of *S. mutans* UM Δ*mucD*. (**b**) Construction of MucD-C domain point mutations. The targeted mutated amino acids are colored in red. (**c**) Construction of *S. mutans* UM Δ*mucE*. (**d**) Construction of *S. mutans* UM Δ*mucF–J*. The homologous arms flanking the targeted gene (colored in grey) for recombination are colored in orange and purple, respectively; IFDC2, IFDC2 cassette as described in Methods.

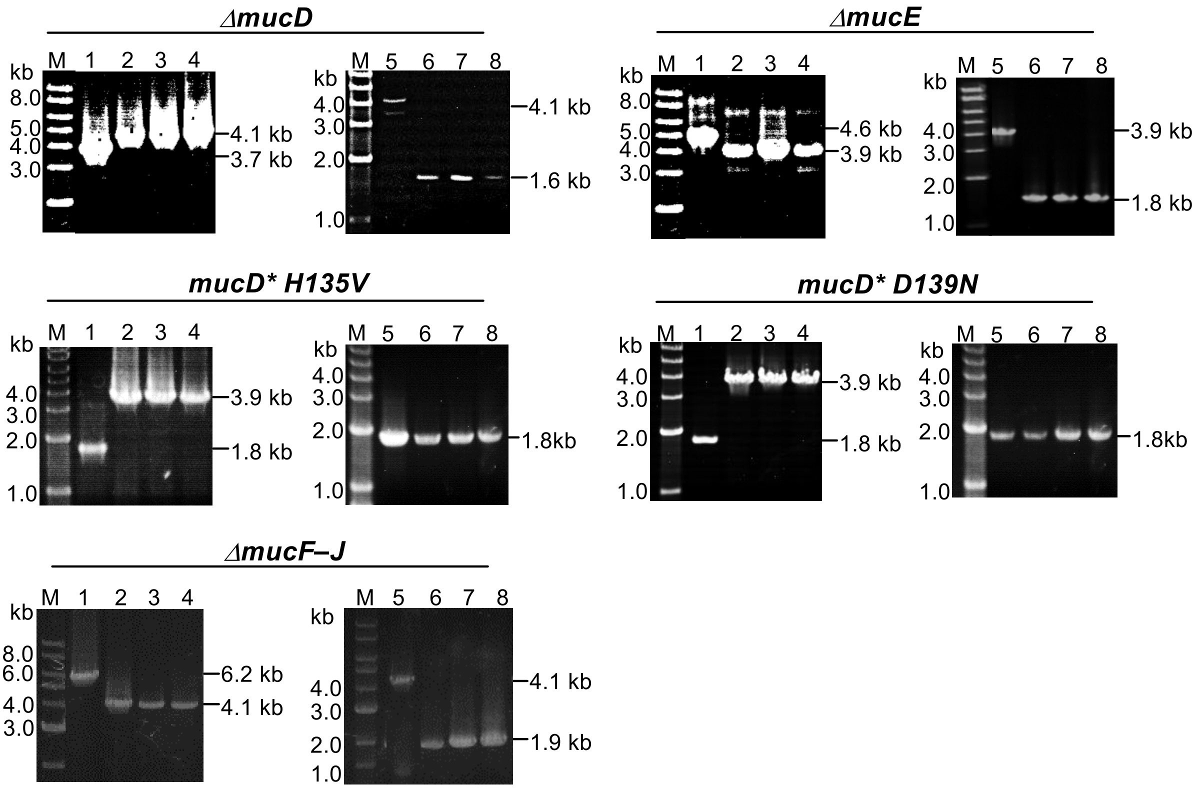

Fig. S5. PCR verifications of *S. mutans* UM in-frame deletion or point mutation strains. For all the mutants: Lane M, DNA marker; Lane 1, negative controls using genomic DNAs of *S. mutans* UM as templates; Lane 2-4, PCR verifications of strains with targeted genes replaced by IFDC2 cassette; Lane 5, negative controls using genomic DNAs of *S. mutans* UM with targeted genes replaced by IFDC2 as template (for *mucD*H135V* and *D139N*, using genomic DNA as template); Lane 6-8, PCR verifications of the in-frame deletion or point mutation strains.

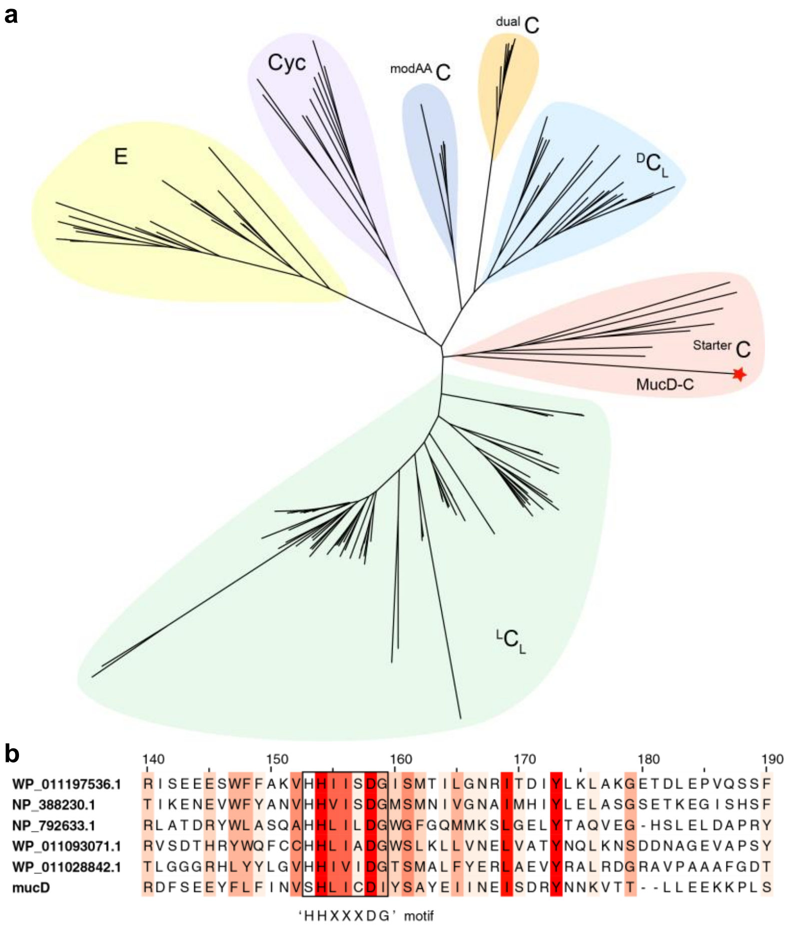

Fig. S6. Unrooted phylogenetic tree of the C-domain superfamily and multiple sequence alignment of MucD-C with starter C domains. (**a**) A total of 142 domains from 21 BGCs are represented with different colors indicate distinct functional clades, including Epimerization (E), Heterocyclization (Cyc), Dehydroamino acid associated (modAAC), Dual epimerization/condensation (DualC), DCL, Starter (StarterC), and LCL. Red star labels the tip for MucD-C. (**b**) The partial result of multiple sequence alignment, black box marks the conserved motif region, in which the essential histidine residue (the second H in motif HHxxxDG) is well conserved.

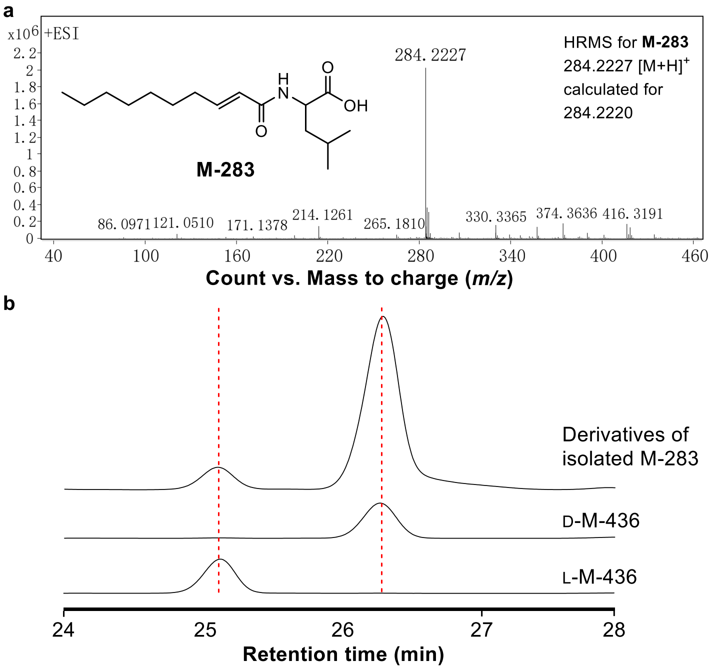

Fig. S7. Characterization of the isolated M-283. (**a**) HRMS spectrum of the isolated **M-283**. (**b**) Comparison of (*S*)-1-(naphthalen-2-yl)ethan-1-amine derivatives of isolated **M-283** with synthetic standards by HPLC. **D-M-436**, (*S*)-1-(naphthalen-2-yl)ethan-1-amine derivative of (*E*)-dec-2-enoyl-D-leucine; **L-M-436**, (*S*)-1-(naphthalen-2-yl)ethan-1-amine derivative of (*E*)-dec-2-enoyl-L-leucine. Derivatives of isolated **M-283** was observed as the mixture of compound **D/L-M-436** in a 9:1 ratio.

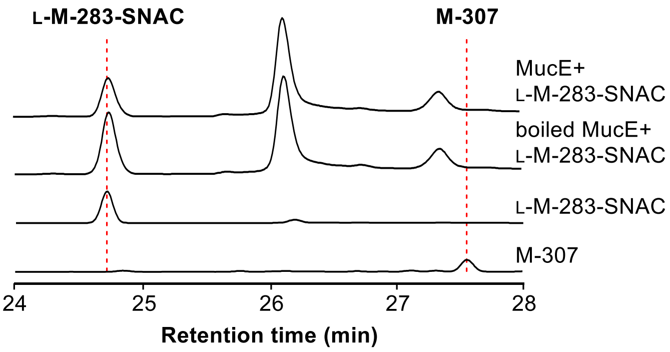

Fig. S8. HPLC analysis of the MucE assays using L-M-283-SNAC as a substrate.

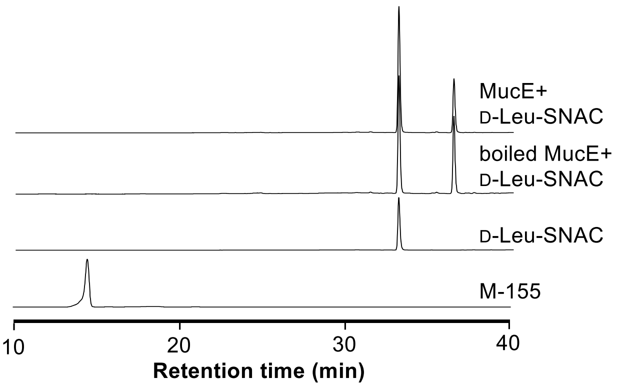

Fig. S9. HPLC analysis of the MucE assays using D-Leu-SNAC as a substrate.

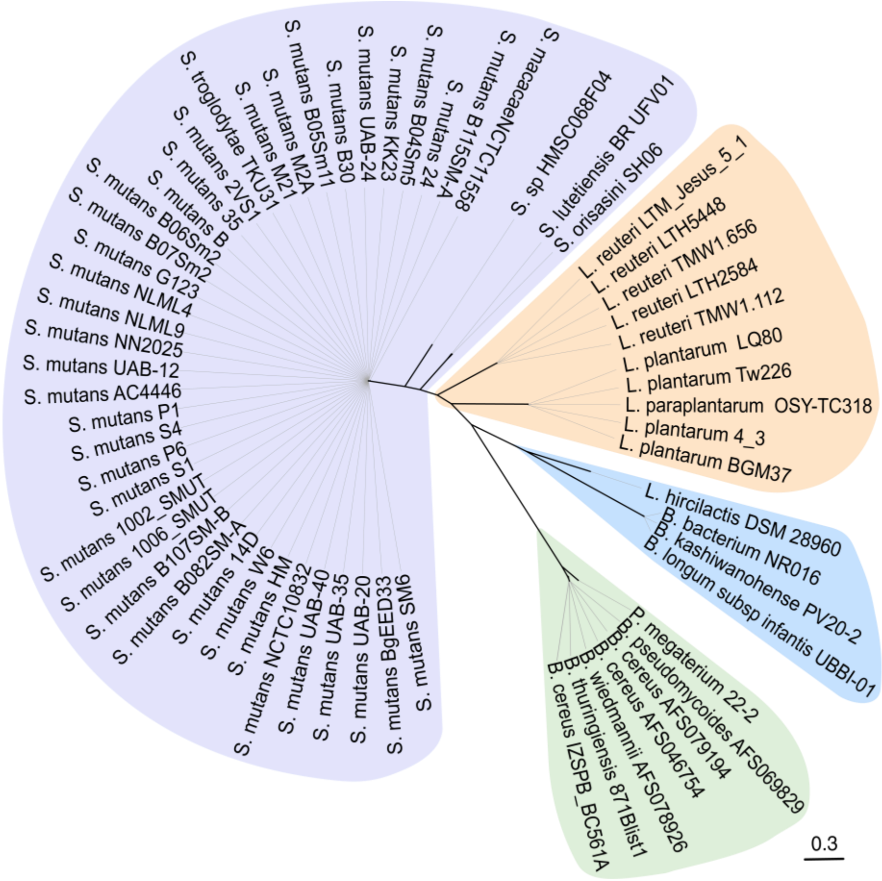

Fig. S10. A detailed phylogenetic tree of the 63 *muc*-like BGCs. The Maximum likelihood tree of nucleotide sequences covering *mucA-E* from the 63 *muc*-like BGCs. Interestingly, all strains from the genus *Bacillus* (green shade) belong to the *B. cereus* sub-clade that can produce diarrheal toxins.

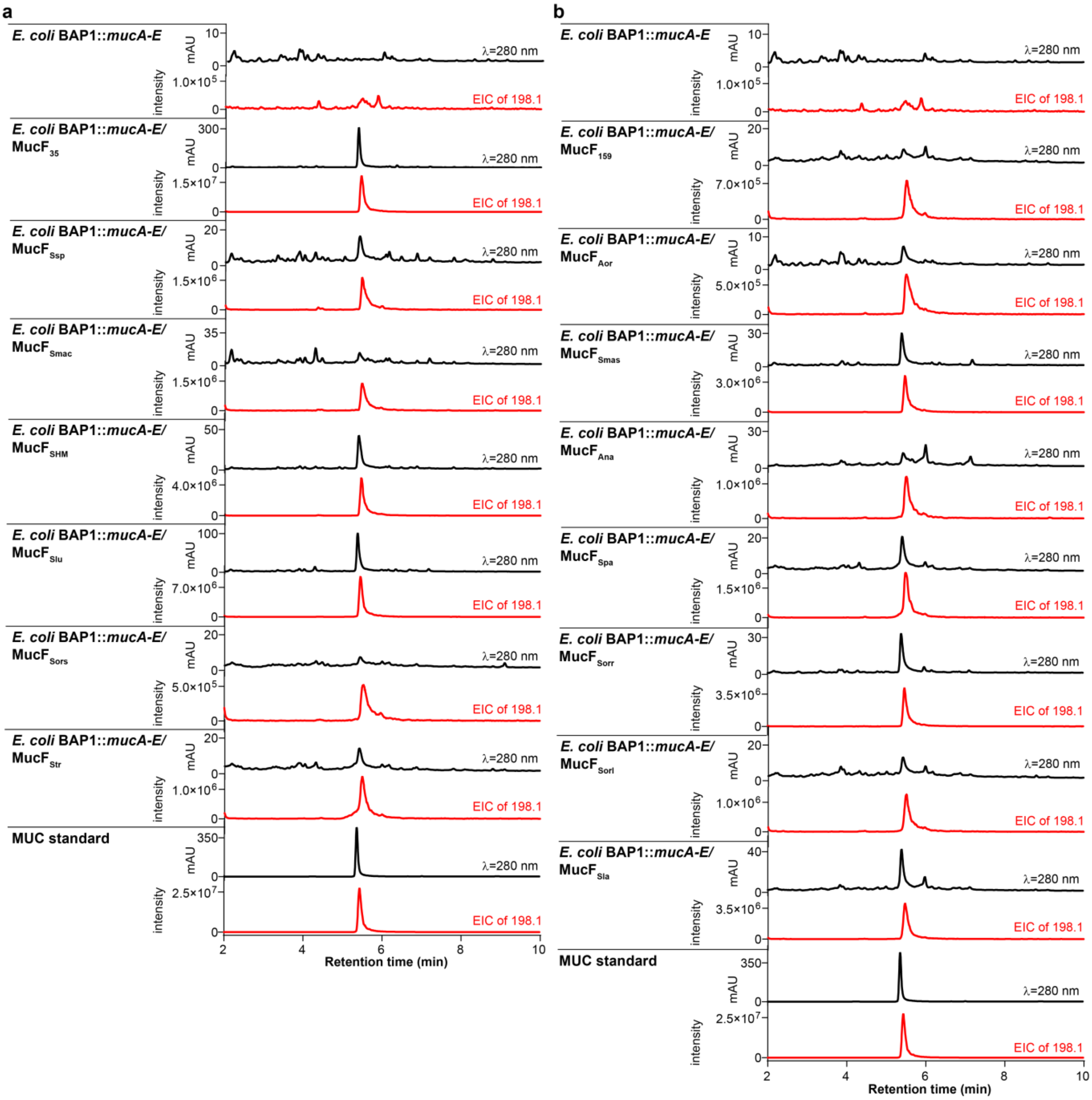

Fig. S11. ***In vivo*** **characterization of the MucF homologous proteins.** (**a**) LC-MS analysis of $MucF_{35}$ and MucF homologous proteins from strains containing *muc* BGC in converting RTC to MUC; (**b**) LC-MS analysis of MucF homologous proteins from strains without *muc* BGC in converting RTC to MUC. MUC was detected.

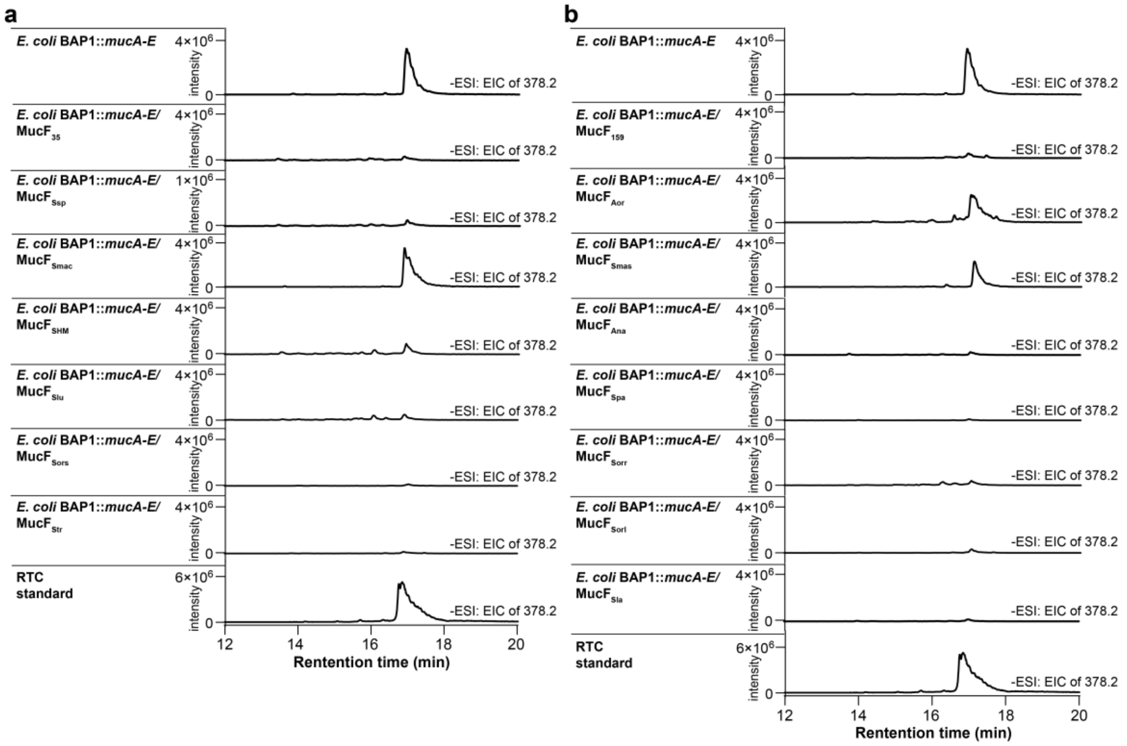

Fig. S12. *In vivo* characterization of the MucF homologous proteins. (**a**) LC-MS analysis of $MucF_{35}$ and MucF homologous proteins from strains containing *muc* BGC in converting RTC to MUC; (**b**) LC-MS analysis of MucF homologous proteins from strains without *muc* BGC in converting RTC to MUC. RTC was detected.

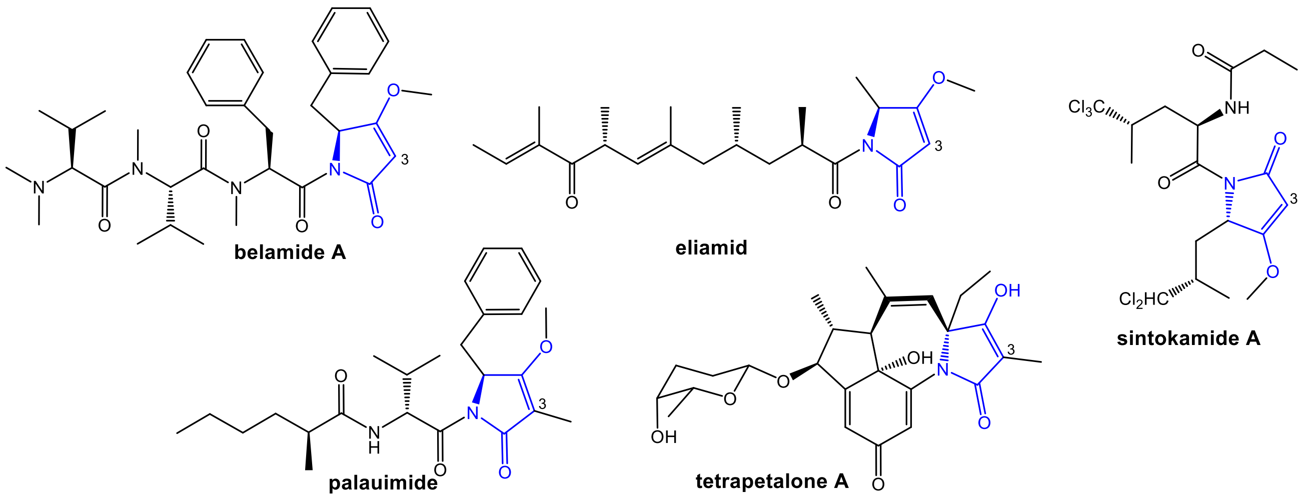

Fig. S13. Structures of some natural TACs lacking C-3 acyl group.

Experimental procedures

1. Synthesis of **D-M-283** and **L-M-283**.

1.1 Synthesis of (*E*)-dec-2-enoyl chloride.

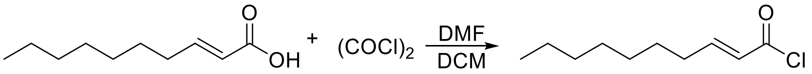

To a solution of (*E*)-dec-2-enoic acid (0.1 g, 1.43 mmol) in dry CH_2Cl_2 (2 mL), oxalyl chloride (0.1 mL, 1.2 mmol) was added dropwise over 10 min, and then 3 drops of DMF were added. After the evolution of gas ceases and the resulting solution was stirred at room temperature for an additional 2 h, the solvent and excess oxalyl chloride were removed to afford the (*E*)-dec-2-enoyl chloride as the crude oil which directly used in the next step without purification.

1.2 Synthesis of (*E*)-dec-2-enoyl-D-leucine (**D-M-283**)

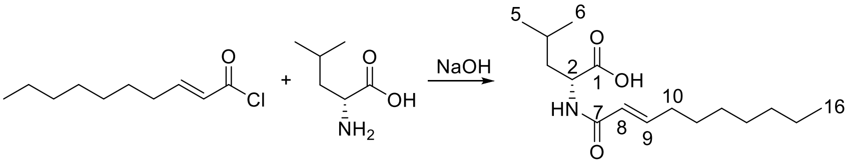

D-M-283

(*E*)-dec-2-enoyl chloride (0.1 g, 0.53 mmol) was added dropwise to a solution of D-leu (0.06 g, 0.46 mmol) dissolved in 2N NaOH (2 mL) and kept at 0°C, the mixture was stirred for 20 min at room temperature. During the reaction the pH of the solution should above 7. After the reaction was complete, the solution was cooled to 0°C and neutralized to 7 with concentrated hydrochloric acid. After extracted with CH_2Cl_2, the organic layer was dried with Na_2SO_4, filtered, and concentrated under reduced pressure, and then purified by column chromatography on silica gel to afford (*E*)-dec-2-enoyl-D-leucine (**D-M-283**) (0.1 g, 66%). **^{1}H NMR** (500 MHz, $CDCl_3$) δ 6.83 (t, *J* =7.5 Hz, 1H, H-9), 5.84 (d, *J* =15.0 Hz, 1H, H-8), 4.56 (m, 1H, H-2), 2.14 (m, 2H), 1.67-1.27 (m, 13H), 0.90 (m, 9H); **^{13}C NMR** (125 MHz, $CDCl_3$) δ 176.0, 166.5, 146.3, 122.8, 51.0, 41.5, 31.8, 31.7, 31.7, 29.1, 28.2, 24.8, 22.8, 22.6, 22.0, 14.1; **HRESIMS** *m/z* 284.2228 $[M+H]^+$ (calcd for $C_{16}H_{30}NO_3$, 284.2220). **HRESIMS**, **^{1}H** and **^{13}C NMR** spectra, see Fig. S14.

1.3 Synthesis of (*E*)-dec-2-enoyl-L-leucine (**L-M-283**)

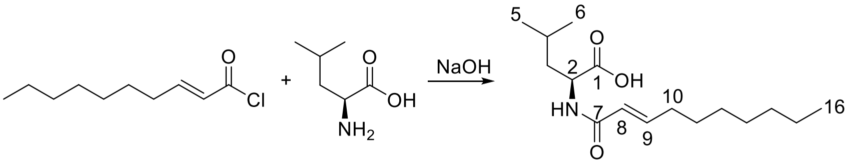

L-M-283

(*E*)-dec-2-enoyl chloride (0.1 g, 0.53 mmol) was added dropwise to a solution of L-leu (0.06 g, 0.46 mmol) dissolved in 2N NaOH (2 mL) and kept at 0°C, the mixture was stirred for 20 min at room temperature. During the reaction the pH of the solution should above 7. After the reaction was complete, the solution was cooled to 0°C and neutralized to 7 with concentrated hydrochloric acid. After extracted with CH_2Cl_2, the organic layer was dried with Na_2SO_4, filtered, and concentrated under reduced pressure, and then purified by column chromatography on silica gel to afford (*E*)-dec-2-enoyl-L-leucine (**L-M-283**) (0.12 g, 79%).**^{1}H NMR** (500 MHz, CD_3OD) δ 6.79 (dt, *J* = 15.0 and 5.0 Hz, 1H, H-9), 6.00 (d, *J* =15.0 Hz, 1H, H-8), 4.46 (dd, *J* = 10.0 and 5.0 Hz, 1H, H-2), 2.20 (m, 2H), 1.71-1.31 (m, 13H), 0.93 (m, 9H); **^{13}C NMR** (125 MHz, CD_3OD) δ 172.9, 167.2, 144.7, 123.1, 51.6, 40.8, 31.6, 31.5, 28.8, 28.8, 28.1, 24.8, 22.3, 22.1, 20.5, 13.0; **ESIMS** *m/z* 284.2 $[M+H]^+$ (calcd for $C_{16}H_{30}NO_3$, 284.2). **ESIMS**, **^{1}H** and **^{13}C NMR** spectra, see Fig. S15.

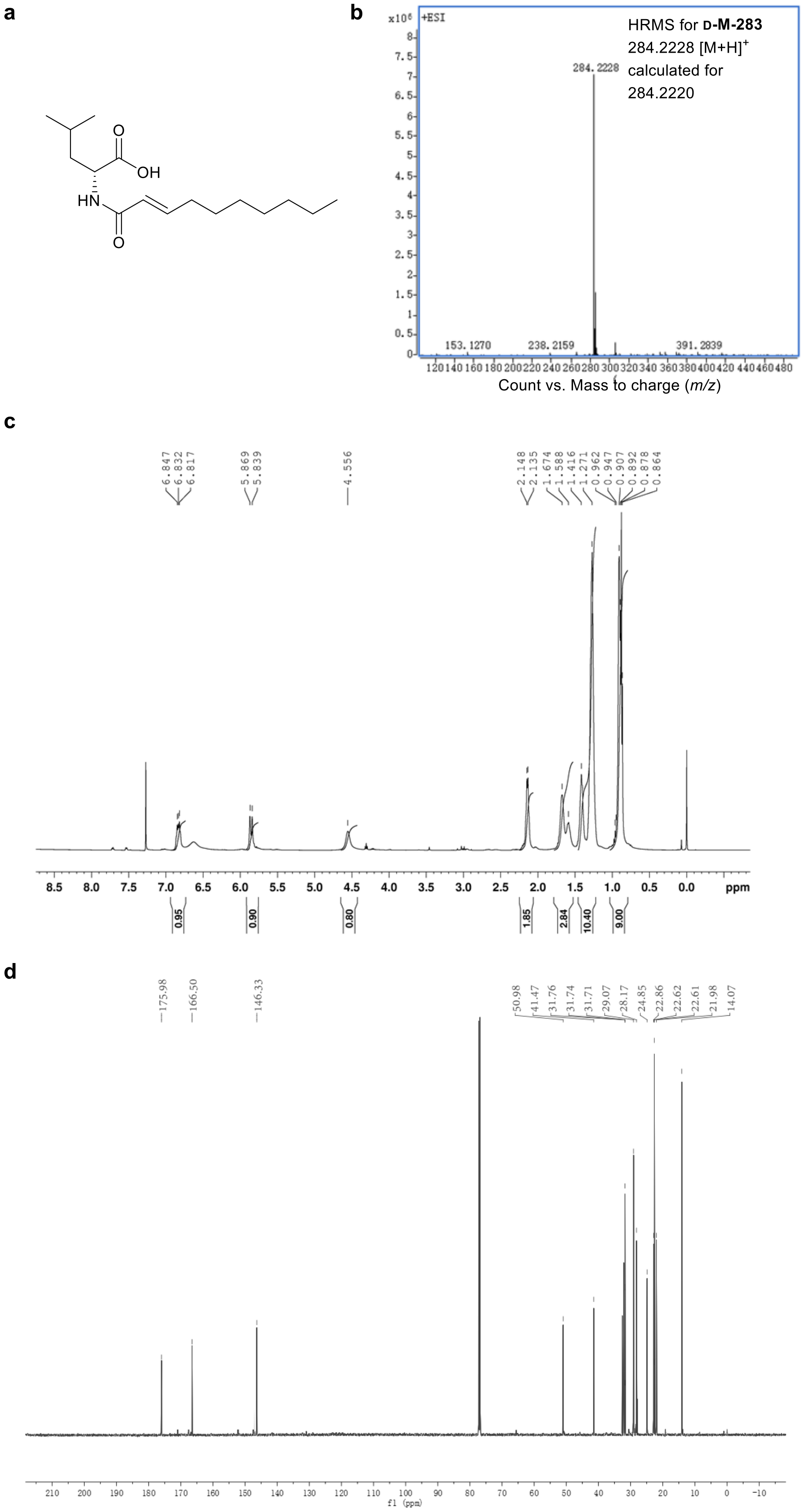

Fig. S14. Spectral data of D-M-283. (**a**) Structure of **D-M-283**; (**b**) HRMS spectrum of **D-M-283**; (**c**) ^{1}H NMR spectrum of **D-M-283**; (**d**) ^{13}C NMR spectrum of **D-M-283**.

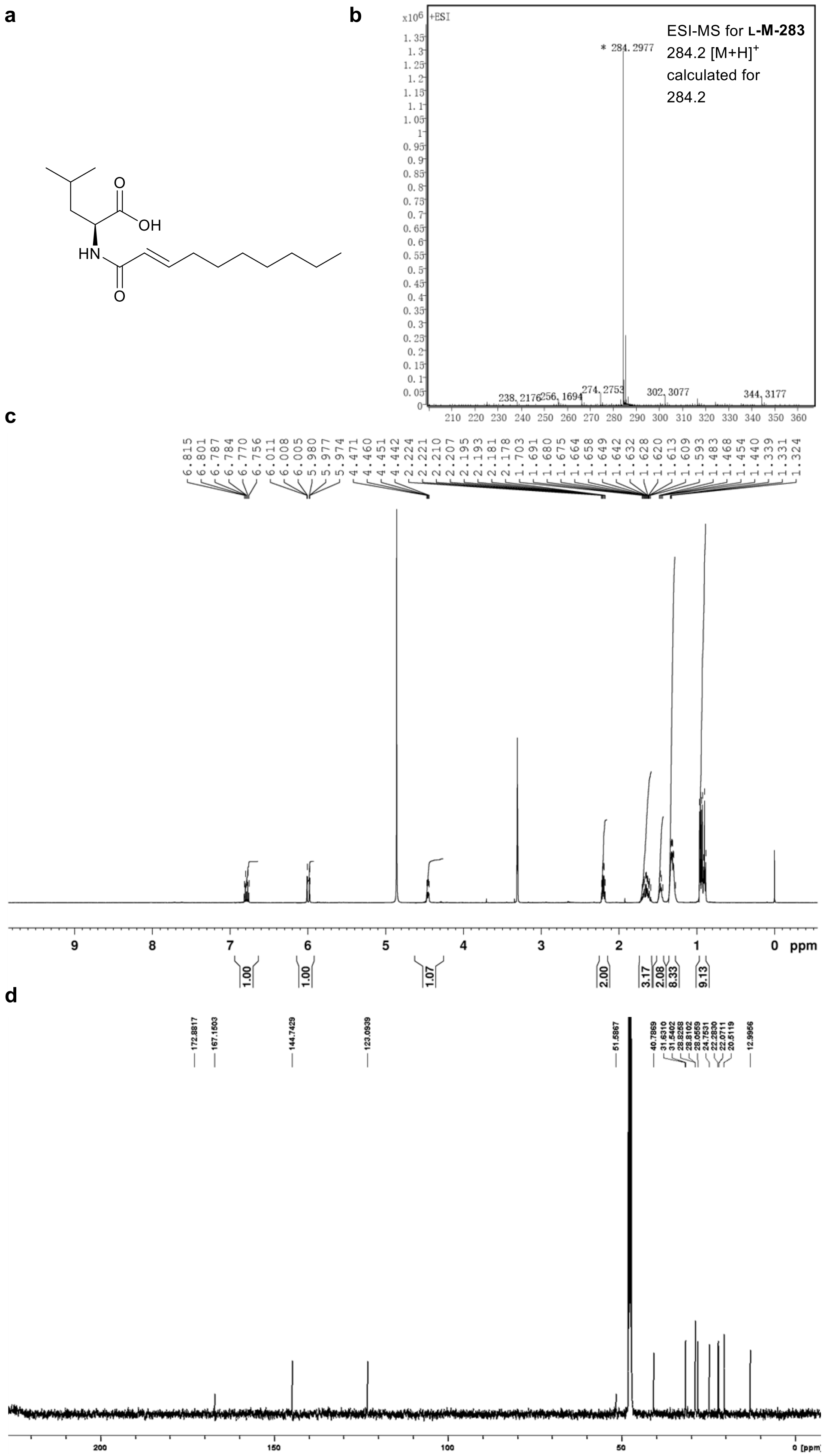

Fig. S15. Spectral data of L-M-283. (**a**) Structure of **L-M-283**; (**b**) MS spectrum of **L-M-283**; (**c**) ^{1}H NMR spectrum of **L-M-283**; (**d**) ^{13}C NMR spectrum of **L-M-283**.

2. Synthesis of **D-M-436** and **L-M-436**.

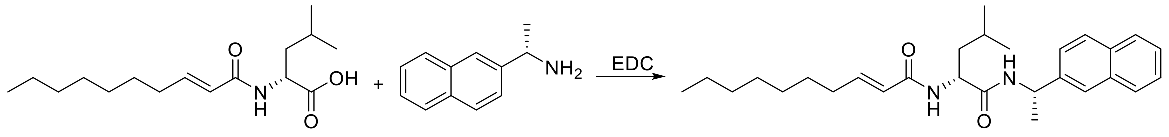

D-M-283 **D-M-436**

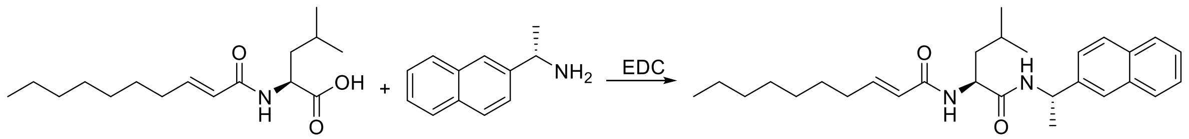

L-M-283 **L-M-436**

EDC (24.5 mg, 0.13mmol) was added to a solution of **D-M-283** or **L-M-283** (10 mg, 0.035 mmol) along with (*S*)-1-(naphthalen-2-yl)ethan-1-amine (12 mg, 0.07mmol) in DCM. The mixture was stirred for 2 h at room temperature and then purified by column chromatography to give (*E*)-N-((*R*)-4-methyl-1-(((*S*)-1-(naphthalen-2-yl)ethyl)amino)-1-oxopentan-2-yl)dec-2-enamide (**D-M-436**) (10 mg, 65.5%). **^{1}H NMR** (500 MHz, $CDCl_3$) δ 8.05 (m, 1H), 7.81 (m, 2H), 7.46 (m, 4H), 6.77 (m, 2H), 5.87 (m, 1H), 4.54 (m, 1H), 2.15 (m, 1H), 1.75 (m, 1H), 1.63 (d, *J* =6.8Hz, 3H), 1.54 (m, 1H), 1.41 (m, 2H), 1.27 (m, 9H), 0.94 (t, *J* =6.7Hz, 3H), 0.88 (t, *J* =6.7Hz, 3H), 0.70 (t, *J* =5.4Hz, 3H). **ESIMS** *m/z* 437.3 $[M+H]^+$ (calcd for $C_{20}H_{37}N_2O_3S$, 437.3); or (*E*)-N-((*S*)-4-methyl-1-(((*S*)-1-(naphthalen-2-yl)ethyl)amino)-1-oxopentan-2-yl)dec-2-enamide (**L-M-436**) (10 mg, 65.5%). **^{1}H NMR** (500 MHz, $CDCl_3$) δ 8.12 (m, 1H), 7.81 (m, 2H), 7.46 (m, 5H), 6.86 (m, 1H), 6.00 (m, 1H), 5.69 (m, 1H), 2.15 (m, 2H), 1.69 (d, *J* =6.8Hz, 3H), 1.51 (m, 1H), 1.41 (m, 2H), 1.27 (m, 9H), 0.87 (m, 9H). **ESIMS** *m/z* 437.3 $[M+H]^+$ (calcd for $C_{20}H_{37}N_2O_3S$, 437.3). **ESIMS** and **^{1}H NMR** spectra, see Fig. S16, S17. (*S*)-1-(naphthalen-2-yl)ethan-1-amine derivatives of isolated **M-283** were prepared similarly and analyzed by HPLC directly after reaction.

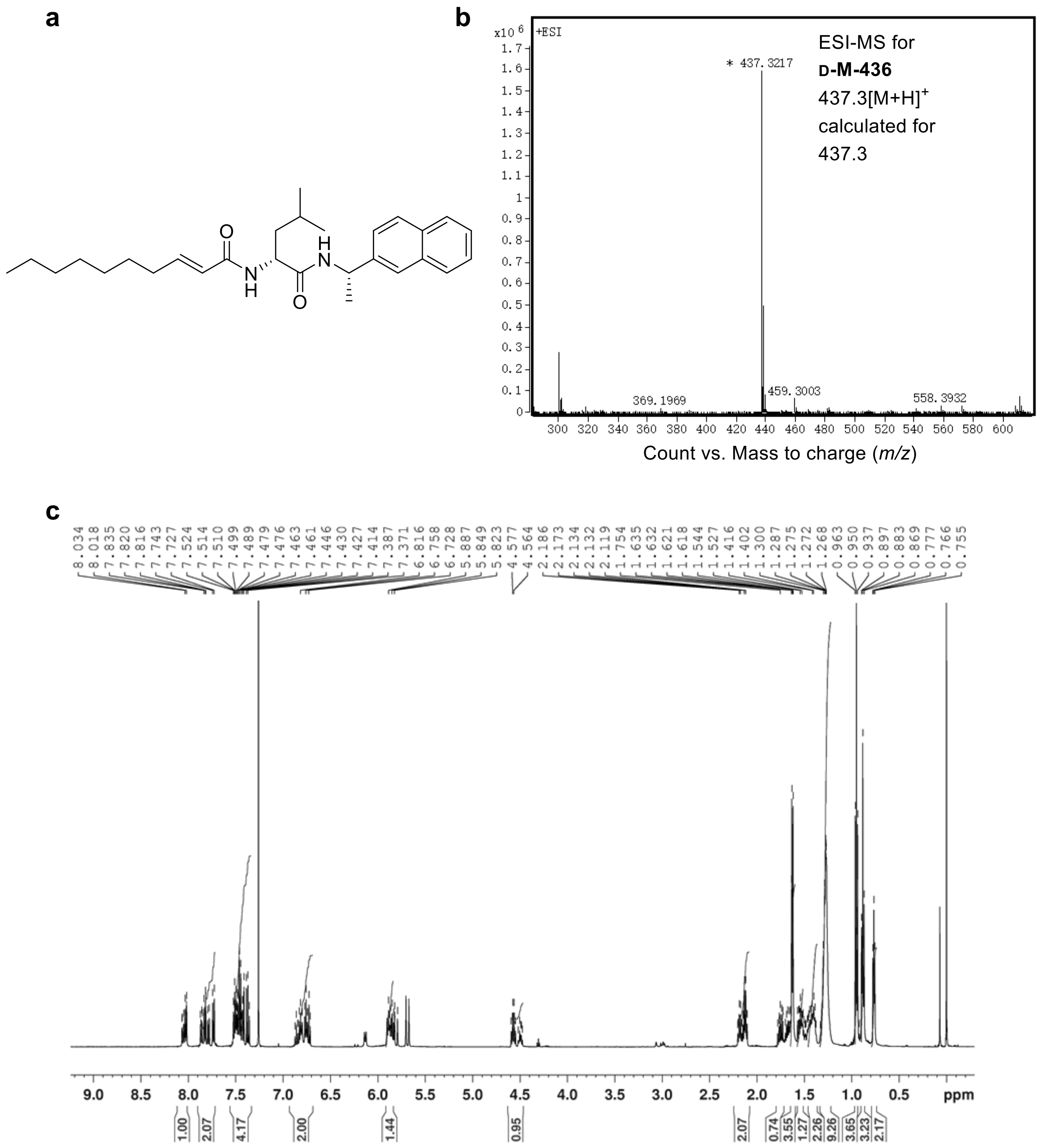

Fig. S16. Spectral data of D-M-436. (**a**) Structure of **D-M-436**; (**b**) MS spectrum of **D-M-436**; (**c**) ^{1}H NMR spectrum of **D-M-436**.

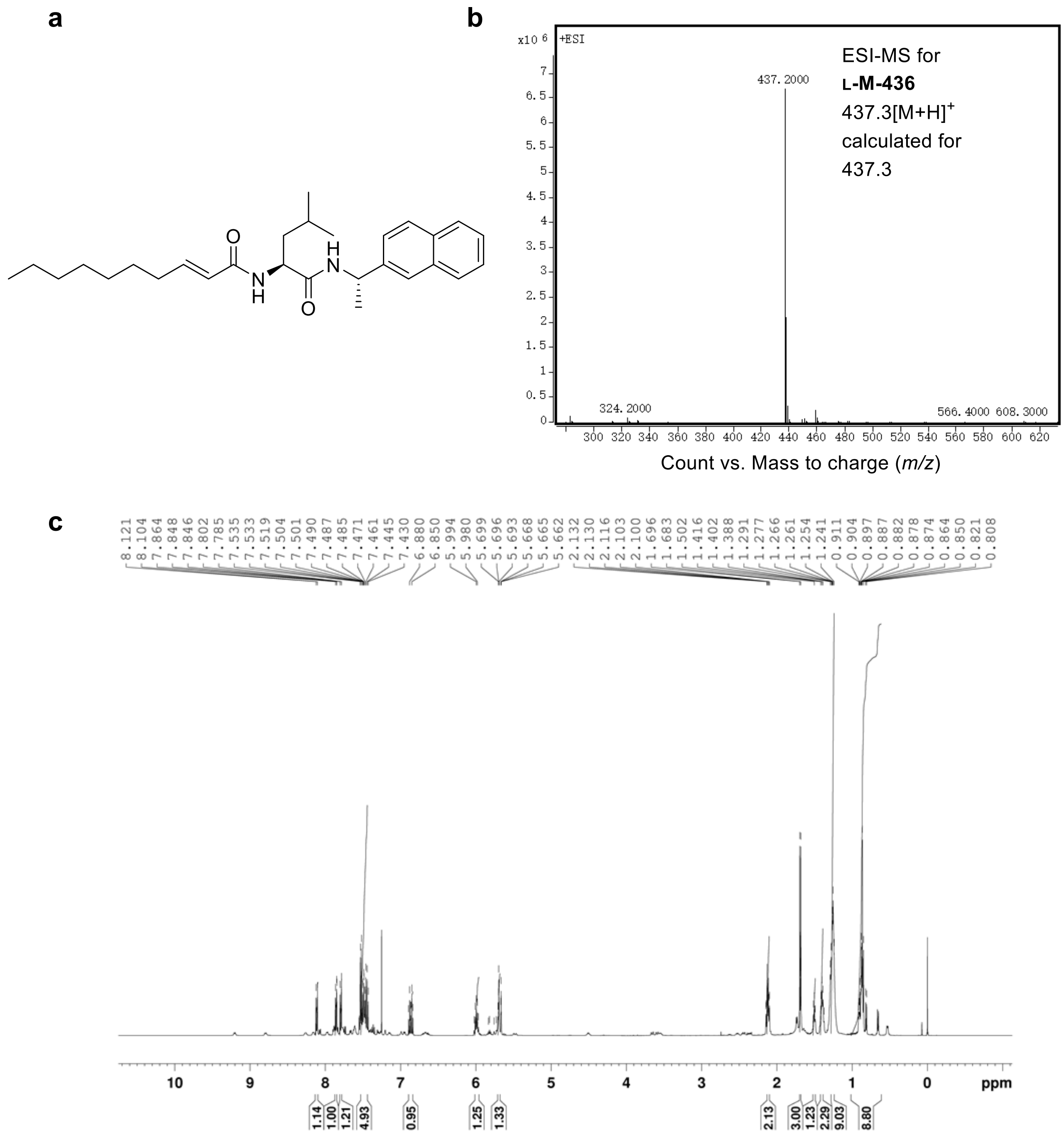

Fig. S17. Spectral data of L-M-436. (**a**) Structure of **L-M-436**; (**b**) MS spectrum of **L-M-436**; (**c**) ^{1}H NMR spectrum of **L-M-436**.

3. Synthesis of **M-307**.

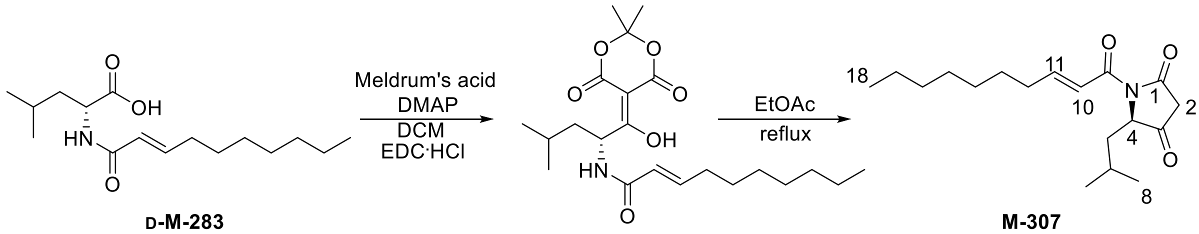

D-M-283 M-307

A solution of Meldrum's acid (0.1 g, 0.69 mmol) and DMAP (0.1 g, 0.82 mmol) in CH_2Cl_2 at 0°C was added with (*E*)-dec-2-enoyl-D-leucine (**D-M-283**) (0.16 g, 0.56 mmol) and then EDC·HCl (0.13 g, 0.68 mmol). The yellow mixture was stirred overnight at room temperature, poured into EtOAc (40 mL), extracted with sulfuric acid (3 × 10 mL, 0.5 M in water) and once with water (20 mL). The layer was dried with Na_2SO_4, filtered, and refluxed until gas formation ceased. All volatiles were evaporated and the yellowish oil was purified by column chromatography on silica gel to afford (*R,E*)-1-(dec-2-enoyl)-5-isobutylpyrrolidine-2,4-dione (**M-307**) (0.1 g, 59%). **^{1}H NMR** (500 MHz, CD_3OD) δ 7.30 (d, *J* =15 Hz, 1H, H-10), 7.03 (m, 1H, H-11), 4.66 (dd, *J* = 3 and 6 Hz, 1H, H-4), 2.26 (m, 2H), 1.93-1.30 (m, 15H), 0.95 (m, 9H); **^{13}C NMR** (125 MHz, CD_3OD) δ 203.8, 180.4, 167.4, 149.2, 123.4, 65.6, 43.3, 39.0, 32.6, 31.7, 29.2, 29.1, 29.1, 29.0, 28.3, 28.2, 22.6, 14.1; **HRESIMS** *m/z* 308.2208 $[M+H]^+$ (calcd for $C_{18}H_{30}NO_3$, 308.2220). **HRESIMS**, **^{1}H** and **^{13}C NMR** spectra, see Fig. S18.

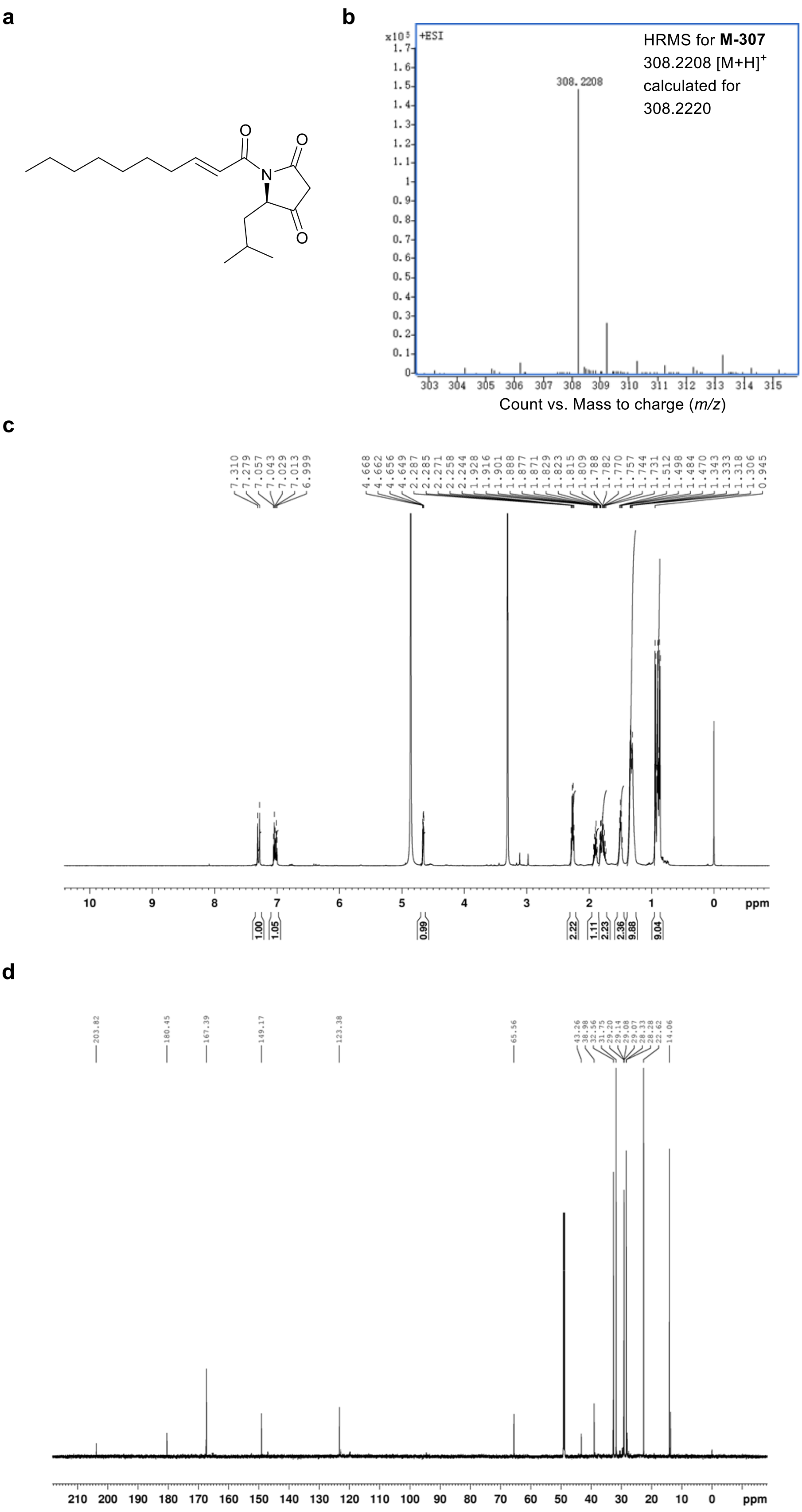

Fig. S18. Spectral data of M-307. (**a**) Structure of **M-307**; (**b**) HRMS spectrum of **M-307**; (**c**) ^{1}H NMR spectrum of **M-307**; (**d**) ^{13}C NMR spectrum of **M-307**.

4. Synthesis of **M-283-SNAC**.

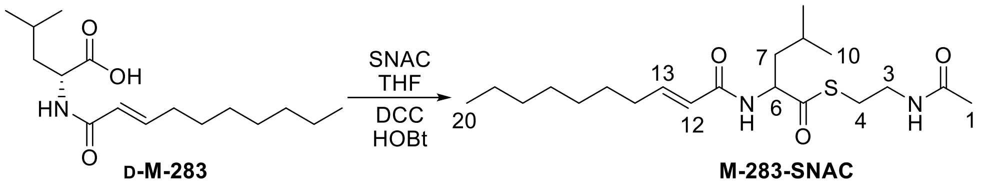

D-M-283 M-283-SNAC

To a solution of (*E*)-dec-2-enoyl-D-leucine (**D-M-283**) (0.05 g, 0.18 mmol) in THF (3 mL), DCC (0.05 g, 0.24 mmol), HOBt (0.07 g, 0.52 mmol), and *N*-acetylcysteamine were added (0.04 g, 0.34 mmol). After the reaction was stirred for 30 min at room temperature, potassium carbonate (0.03 g, 0.22 mmol) was added, and the reaction was stirred for a further 2.5 h. After extracted with CH_2Cl_2, the organic layer was dried with Na_2SO_4, filtered, and concentrated under reduced pressure, and then purified by column chromatography on silica gel to afford *S*-(2-acetamidoethyl) (*E*)-2-(dec-2-enamido)-4-methylpentanethioate (**M-283-SNAC**) (0.03 g, 51%). **^{1}H NMR** (500 MHz, CD_3OD) δ 6.87 (q, *J* =14 Hz, 1H, H-13), 6.01 (d, *J* =15 Hz, 1H, H-12), 4.60 (m, 1H, H-6), 2.99 (t, *J* = 6.6 Hz, 2H, H-3), 2.50 (q, *J* = 13.5 Hz, 2H, H-4), 1.92 (s, 3H, H-1), 1.80-1.36 (m, 13H), 0.95 (m, 9H); **^{13}C NMR** (125 MHz, CD_3OD) δ 203.8, 180.5, 165.1, 147.0, 119.8, 58.8, 43.2, 37.5, 35.8, 32.6, 31.7, 29.2, 29.1, 29.1, 29.1, 29.0, 28.3, 28.2, 22.7, 14.1; **HRESIMS** *m/z* 385.2512 $[M+H]^+$ (calcd for $C_{20}H_{37}N_2O_3S$, 385.2519). **HRESIMS**, **^{1}H** and **^{13}C NMR** spectra, see Fig. S19.

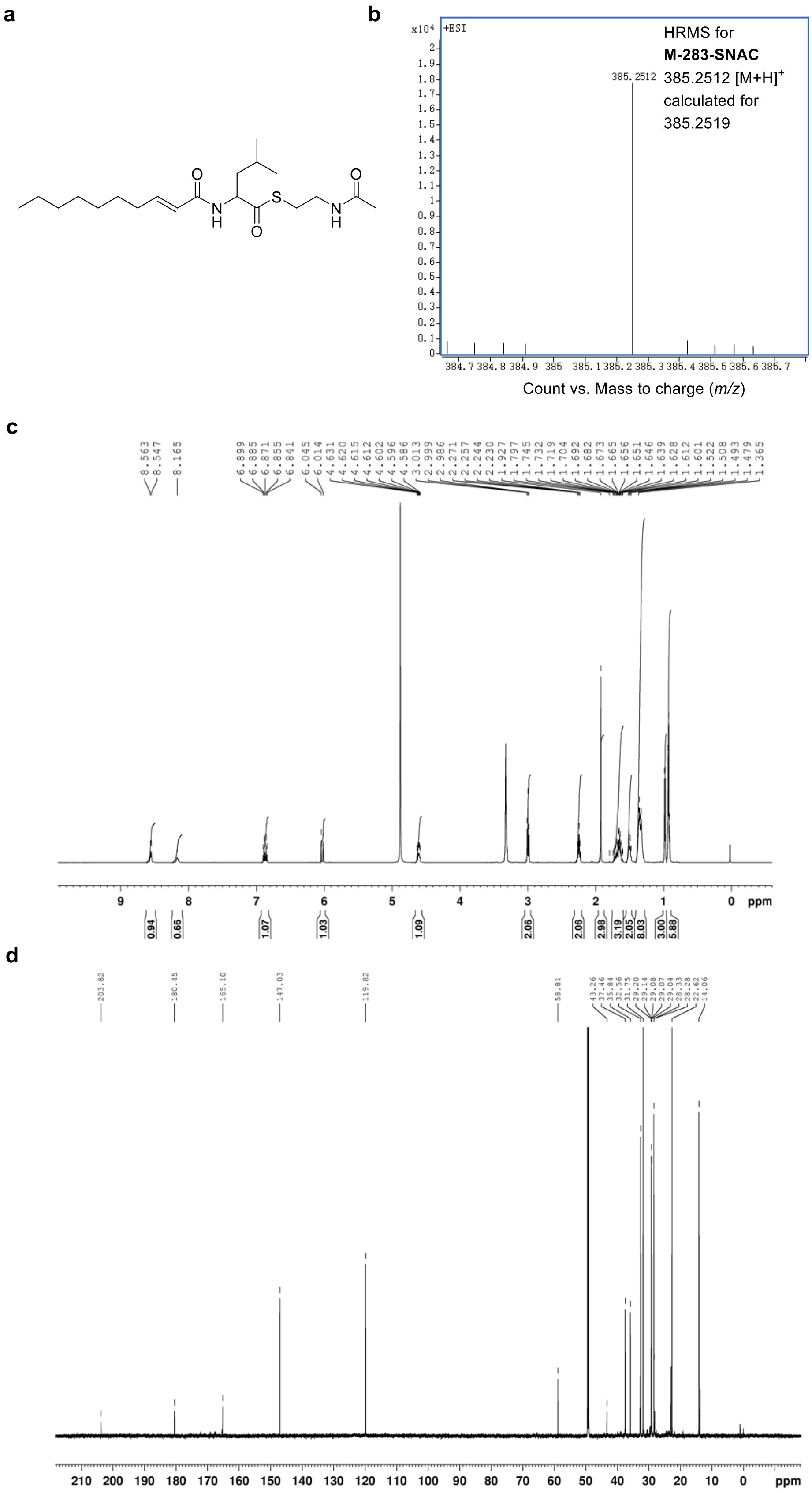

Fig. S19. Spectral data of M-283-SNAC. (**a**) Structure of **M-283-SNAC**; (**b**) HRMS spectrum of **M-283-SNAC**; (**c**) ^{1}H NMR spectrum of **M-283-SNAC**; (**d**) ^{13}C NMR spectrum of **M-283-SNAC**.

5. Synthesis of **D-Leu-SNAC**.

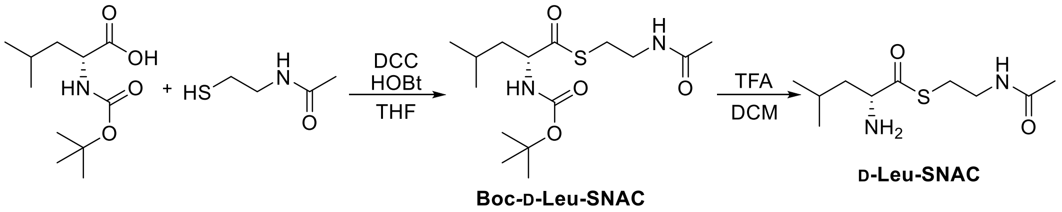

To a solution of Boc-D-Leu (0.05 g, 0.22 mmol) in THF (3 mL), DCC (0.06 g, 0.24 mmol), HOBt (0.08 g, 0.62 mmol), and N-acetylcysteamine were added (0.05 g, 0.41 mmol), after the reaction was stirred for 30 min at room temperature, potassium carbonate (0.04 g, 0.26 mmol) was then added, and the reaction stirred for 2.5 h. The solution was extracted with EtOAc. The organic layer was dried with Na_2SO_4, filtered and concentrated under reduced pressure, and then purified by column chromatography on silica gel to afford *S*-(2-acetamidoethyl) (*R*)-2-((tert-butoxycarbonyl)amino)-4-methylpentanethioate (**Boc-D-Leu-SNAC**) (0.04 g, 57%). Deprotection was carried out by dissolving the **Boc-D-Leu-SNAC** (0.04 g, 0.12 mmol) in 2 mL of 50% TFA/CH_2Cl_2. the solution was stirred at room temperature for 1 h, the mixture was concentrated by rotary evaporation. The mixture was taken up in CH_2Cl_2 and concentrated under reduced pressure, then purified by column chromatography on silica gel to yield **D-Leu-SNAC** (0.02g, 65%). **^{1}H NMR** (500 MHz, CD_3OD) δ 3.97 (dd, *J* = 8.0 and 6.0 Hz, 1H, H-2), 3.13 (t, *J* = 6.8 Hz, 2H), 2.80 (t, *J* = 6.8 Hz, 2H), 2. 10 (s, 3H, H-10), 1.84 (m, 2H, H-3), 1.70 (m, 1H, H-4), 1.00 (m, 6H); **^{13}C NMR** (125 MHz, CD_3OD) δ 173.9, 170.9, 51.0, 42.1, 39.4, 37.9, 24.2, 21.3, 20.8, 19.4. **ESIMS** *m/z* 233.1 $[M+H]^+$ (calcd for $C_{10}H_{21}N_2O_2S$, 233.1). **ESIMS**, **^{1}H** and **^{13}C NMR** spectra, see Fig. S20.

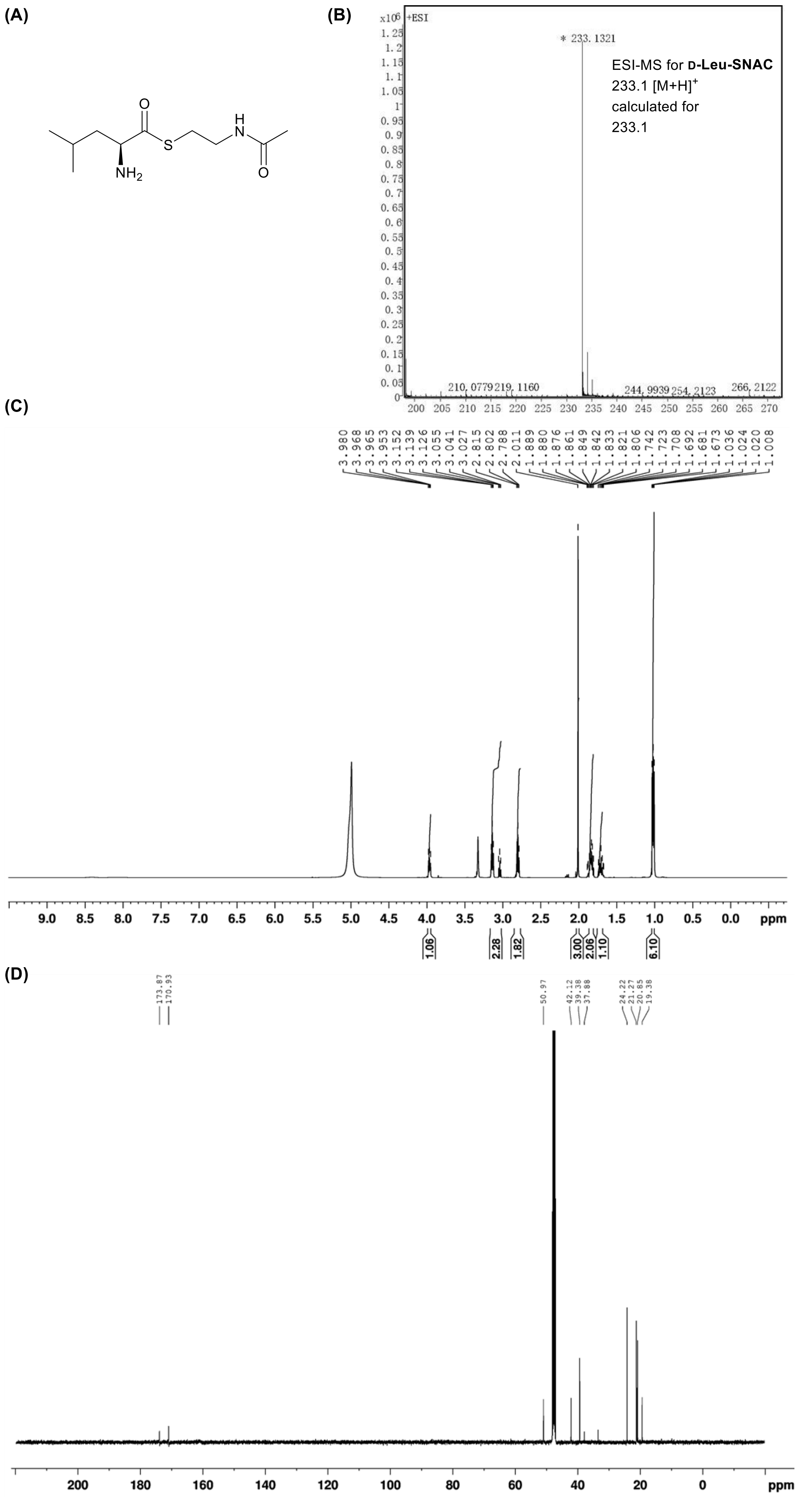

Fig. S20. Spectral data of D-Leu-SNAC. (**a**) Structure of **D-Leu-SNAC**; (**b**) MS spectrum of **D-Leu-SNAC**; (**c**) ^{1}H NMR spectrum of **D-Leu-SNAC**; (**d**) ^{13}C NMR spectrum of **D-Leu-SNAC**.

6. Synthesis of **M-155**.

6.1 Synthesis of *tert*-butyl (*R*)-2-isobutyl-3, 5-dioxopyrrolidine-1-carboxylate.

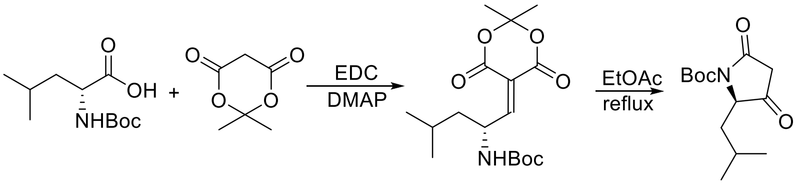

To a stirred solution of Boc-D-Leu (0.5 g, 2.16 mmol) in dry CH_2Cl_2 (5 mL) at room temperature, Meldrum's acid (0.34 g, 2.37 mmol), DMAP (0.37 g, 3.02 mmol) and EDC·HCl (suspended in 4 mL CH_2Cl_2, 0.5 g, 2.59 mmol) were added. After 1.5 h the solution was concentrated under reduced pressure, dissolved in EtOAc (200 mL), extracted thrice with sulfuric acid (3 x 50 mL, 0.5 M in water) and once with water (100 mL). The organic phase was dried with Na_2SO_4, filtered, and concentrated under reduced pressure. The crude product was redissolved in EtOAc and refluxed until gas formation ceased. Then all volatiles were evaporated and the yellowish oil was purified by column chromatography on silica gel to afford *tert*-butyl (*R*)-2-isobutyl-3,5-dioxopyrrolidine-1-carboxylate (0.4 g, 73%).

6.2 Synthesis of (*R*)-5-isobutylpyrrolidine-2,4-dione (**M-155**).

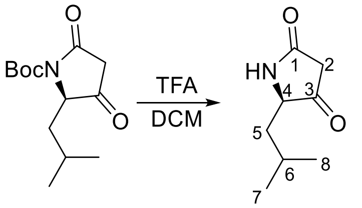

M-155

tert-butyl (*R*)-2-isobutyl-3,5-dioxopyrrolidine-1-carboxylate (0.2 g, 0.78 mmol) was then dissolved in mixture of CH_2Cl_2 (2 ml) and TFA (2 ml). The mixture was stirred for 30 min. After completion of the reaction, the solution was extracted with CH_2Cl_2 and 5 % aqueous HCl. The organic layer was dried with Na_2SO_4, filtered, and concentrated under reduced pressure. The remaining oil was purified by column chromatography on silica gel to afford (*R*)-5-isobutylpyrrolidine-2,4-dione (**M-155**) (0.1 g, 83 %). **^{1}H NMR** (500 MHz, $CDCl_3$) δ 4.05 (dd, *J* = 4.4 and 9.6 Hz, 1H, H-4), 3.05 (s, 2H, H-2), 1.80, 1.65, 1.50 (m, 3H), 0.97 (t, *J* = 6.6 Hz, 6H, H-7,8); **^{13}C NMR** (125 MHz, $CDCl_3$) δ 199.6, 172.6, 59.2, 41.6, 25.2, 23.9, 23.3, 21.6; **HRESIMS** *m/z* 156.1032 $[M+H]^+$ (calcd for $C_8H_{14}NO_2$, 156.1019). **HRESIMS**, **^{1}H** and **^{13}C NMR** spectra, see Fig. S21.

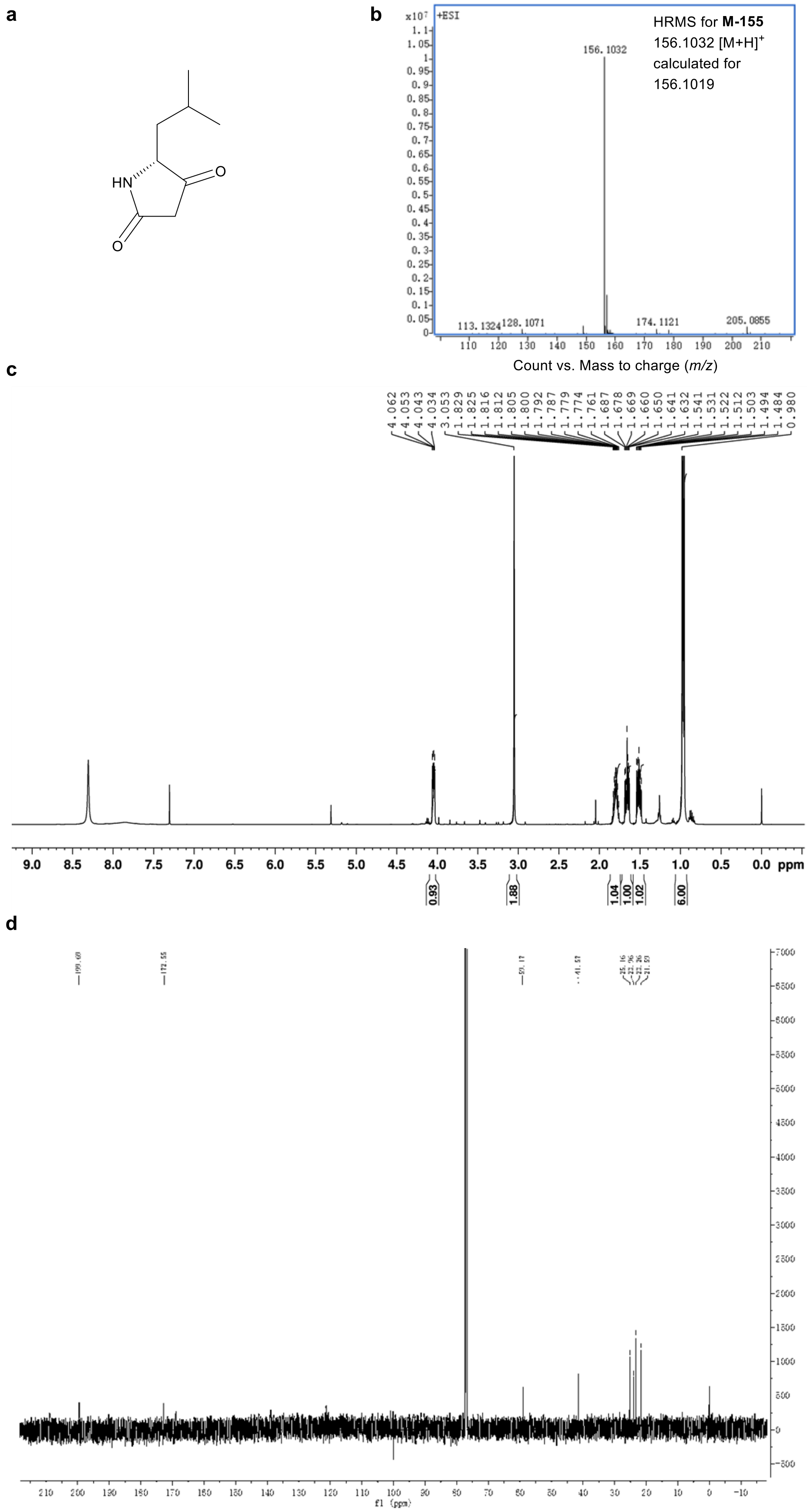

Fig. S21. Spectral data of M-155. (**a**) Structure of **M-155**; (**b**) HRMS spectrum of **M-155**; (**c**) ^{1}H NMR spectrum of **M-155**; (**d**) ^{13}C NMR spectrum of **M-155**.

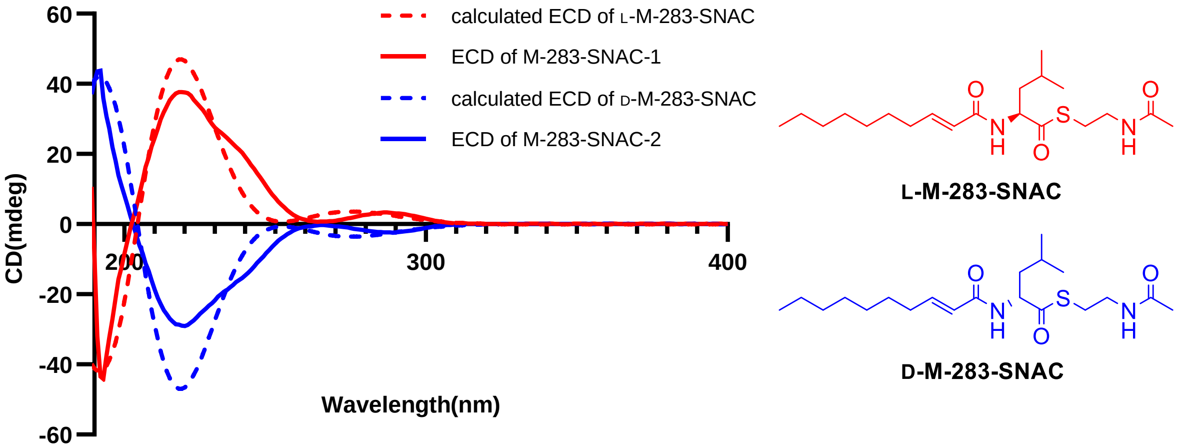

Fig. S22. Comparison of the experimental ECD spectra of M-283-SNAC-1, M-283-SNAC-2 and the calculated ECD spectra of L-M-283-SNAC and D-M-283-SNAC.

Table S3 (separate file). BLASTP result of 111 MucF homologous proteins matching $MucF_{35}$ (tab 1); detail information about $MucF_{35}$ and 17 MucF homologues used in the activity testing (tab 2).

References

1 Hao, T. *et al.* An anaerobic bacterium host system for heterologous expression of natural product biosynthetic gene clusters. *Nat. Commun.* **10**, 1-13 (2019).

2 Qi, F., Chen, P. & Caufield, P. W. The group I strain of *Streptococcus mutans*, UA140, produces both the lantibiotic mutacin I and a nonlantibiotic bacteriocin, mutacin IV. *Appl. Environ. Microbiol.* **67**, 15-21 (2001).

3 Belda, E. *et al.* An updated metabolic view of the *Bacillus subtilis* 168 genome. *Microbiology* **159**, 757-770 (2013).

4 Pfeifer, B. A., Admiraal, S. J., Gramajo, H., Cane, D. E. & Khosla, C. Biosynthesis of complex polyketides in a metabolically engineered strain of *E. coli*. *Science* **291**, 1790-1792 (2001).

5 Xie, Z., Okinaga, T., Qi, F., Zhang, Z. & Merritt, J. Cloning-independent and counterselectable markerless mutagenesis system in *Streptococcus mutans*. *Appl. Environ. Microbiol.* **77**, 8025-8033 (2011)

6 Xie, Z., Qi, F. & Merritt, J. Development of a tunable wide-range gene induction system useful for the study of streptococcal toxin-antitoxin systems. *Appl. Environ. Microbiol.* **79**, 6375-6384 (2013).

7 Tang, X. *et al.* Cariogenic *Streptococcus mutans* produces tetramic acid strain-specific antibiotics that impair commensal colonization. *ACS Infect. Dis.* **6**, 563–571 (2020).

8 Liu, X. & Walsh, C. T. Cyclopiazonic acid biosynthesis in *Aspergillus* sp.: characterization of a reductase-like R* domain in cyclopiazonate synthetase that forms and releases cyclo-acetoacetyl-L-tryptophan. *Biochemistry* **48**, 8746-8757 (2009).

9 Kato, N. *et al.* Genetic safeguard against mycotoxin cyclopiazonic acid production in *Aspergillus oryzae*. *ChemBioChem* **12**, 1376–1382 (2011).

10 Tokuoka, M. *et al.* Cyclopiazonic acid biosynthesis gene cluster gene *cpaM* is required for speradine A biosynthesis. *Biosci. Biotechnol. Biochem.* **79**, 2081-2085 (2015).
